# The grey water footprint of human and veterinary pharmaceuticals

**DOI:** 10.1016/j.wroa.2020.100044

**Published:** 2020-01-16

**Authors:** Lara Wöhler, Gunnar Niebaum, Maarten Krol, Arjen Y. Hoekstra

**Affiliations:** a Twente Water Centre, Faculty of Engineering Technology, University of Twente, Horst Complex Z223, P.O Box 217, 7500, AE, Enschede, Netherlands; b Institute of Environmental Systems Research, Osnabrück University, Barbarastraße 12, D-49076, Osnabrück, Germany; c Institute of Water Policy, Lee Kuan Yew School of Public Policy, National University of Singapore, 469C Bukit Timah Road, 259772, Singapore

**Keywords:** Grey water footprint, Water pollution, Pharmaceuticals, Human health, Livestock, Manure

## Abstract

Water pollution by pharmaceuticals is widespread, causing both environmental and human health risks. We assess pharmaceutical water pollution from human and veterinary pharmaceuticals at three geographical levels: global, national (considering Germany and the Netherlands) and catchment level (with a case study for the Vecht catchment shared by Germany and the Netherlands). The grey water footprint (GWF), a measure of water pollution in volumetric terms, is estimated from pharmaceutical loads entering the aquatic environment, considering different pollutant sources and pathways. We study different substances depending on data availability, which varies across geographical levels. Results show a global per capita GWF of 1900 m^3^ yr^−1^ resulting from human consumption of ciprofloxacin. The largest GWFs in both Germany and the Netherlands were found for ethinylestradiol for human and amoxicillin for veterinary use. The estimated per capita GWF from human use of ethinylestradiol is 2300 m^3^ yr^−1^ for Germany and 11,300 m^3^ yr^−1^ for the Netherlands. The per capita GWFs of German and Dutch consumers of animal products are 12,900 and 10,600 m^3^ yr^−1^, respectively. For the Vecht catchment, we estimate the water pollution level per sub-catchment by comparing the GWF to available runoff, which enables us to identify geographic hotspots. In the basin as a whole, GWFs from human and veterinary pharmaceuticals both exceed available runoff. At all levels, pharmaceutical water pollution substantially adds to earlier water footprint studies that excluded this type of pollution, which demonstrates the importance to include pharmaceutics in water footprint studies.

## Highlights

• This is the first broad study on grey water footprint (GWF) of pharmaceuticals.
• Human and veterinary compounds are included and three spatial levels assessed.
• GWFs of pharmaceuticals are substantial compared to GWFs of other pollutants.
• Animal products add a substantial part to consumer’s pharmaceutical-related GWF.
• Grey water footprints exceed available runoff in a case study for the Vecht basin.

## Units

kg yr^−1^: kilogram per year
kg m^−3^: kilogram per cubic metre
kg day^−1^: kilogram per day
kg ha^−1^ yr^−1^: kilogram per hectare per year
m^3^ yr^−1^: cubic metre per year
m^3^ animal^−1^: cubic metre per animal
m^3^ km^−2^: cubic metre per square kilometre
μg L^−1^: microgram per litre

## 1 Introduction

**Fig. 1:**
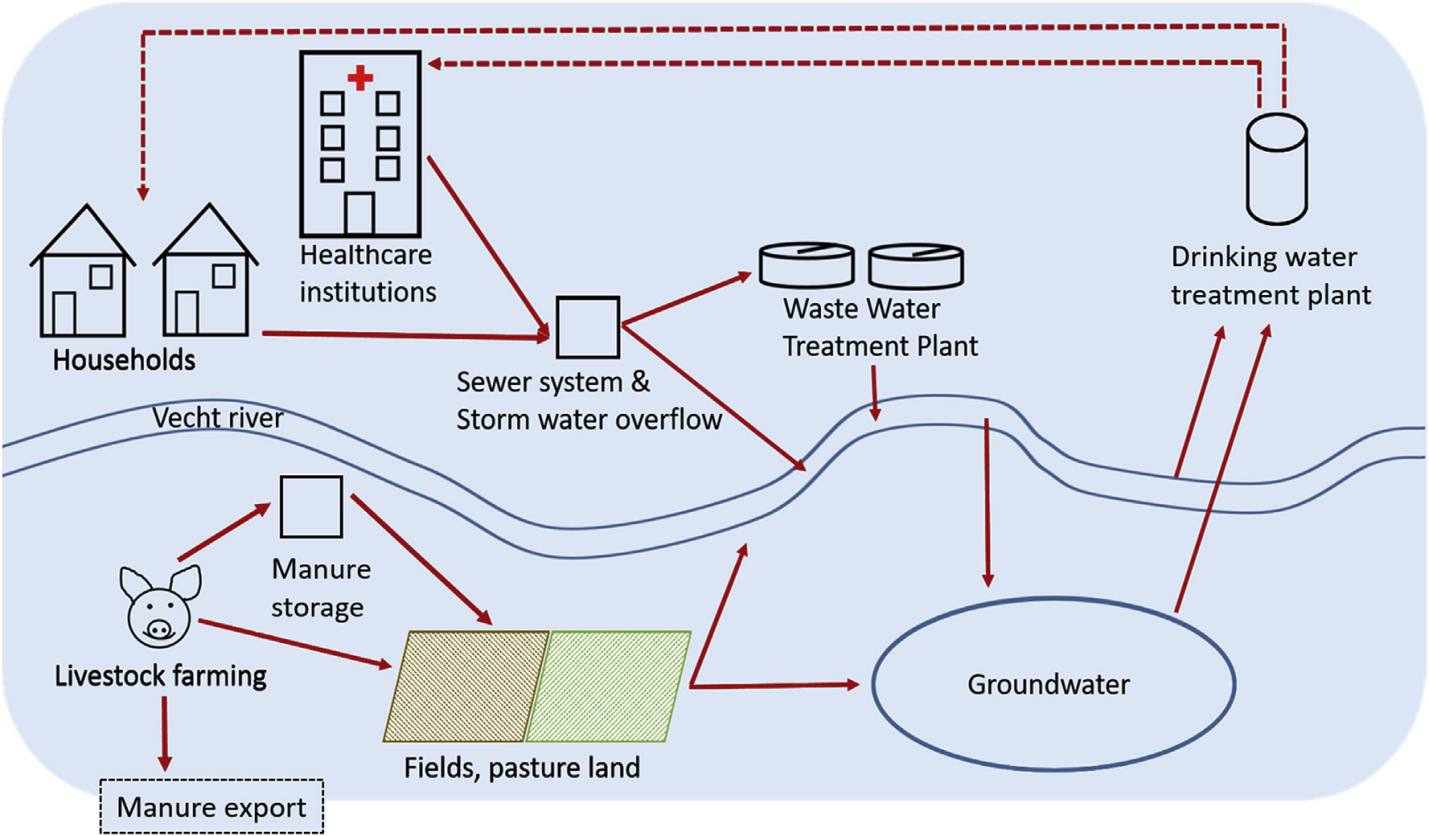
Pathways of human and veterinarian pharmaceuticals entering the environment.

Worldwide, about 600 pharmaceutical compounds and transformation products from pharmaceuticals have been traced in the aquatic environment (Aus der Beek et al., 2015). Exposure to pharmaceuticals has led to ecotoxicological effects on various species, such as vultures, fish, frogs and duckweed (Aus der Beek et al., 2015; Sumpter, 2010), which has resulted in serious concerns, especially regarding drinking water risks (WHO, 2012) and antimicrobial resistance (WHO, 2014). Urban wastewater discharge is generally regarded the dominant source of pharmaceuticals in water, whereas discharge from manufacturing, hospitals, animal husbandry and aquaculture can be important locally (Aus der Beek et al., 2015). Human and veterinary pharmaceuticals enter the aquatic environment via distinct pathways (Kümmerer, 2008a). Fig. 1 illustrates sources and pathways of pharmaceutical residues to freshwater resources considered in this study.

Pharmaceuticals are designed to cure people and animals, or diagnose or prevent diseases. They have a precise function within target bodies. The administered dose is rarely entirely decomposed by the body; substantial fractions are generally excreted, mainly via urine (Winker et al., 2008). The excreted fractions of human pharmaceuticals and their metabolites are mostly discharged into the sewer system (Hughes et al., 2013) and enter the receiving water body as point source. In the case of livestock, manure from treated animals, collected in liquid, solid or mixed form (Weinfurtner, 2011), contains pharmaceutical residues and will generally be applied to crop fields or grasslands as fertilizer (Kümmerer, 2008b). A fraction of the pharmaceuticals thus brought onto land will leach to groundwater or reach open water through surface runoff (Boxall, 2008), thus forming a diffuse source of pollution.

This study uses the grey water footprint (GWF) concept to assess water pollution by pharmaceuticals from households, hospitals and animal husbandry considering different pathways. The GWF indicates the volume of water required to assimilate pollutant loads to acceptable concentrations (Hoekstra et al., 2011). Despite water footprint research since 2002 (Hoekstra, 2017), investigation of the GWF related to pharmaceuticals is in its infancy. There has been extensive research into the GWF related to the use of fertilizers (Liu et al., 2012; Mekonnen and Hoekstra, 2015, 2018) and pesticides (Gil et al., 2017; Lamastra et al., 2014; Vale et al., 2019), but only one case study on the GWF of human pharmaceutical use has been published (Martinez-Alcala et al., 2018). There have been various efforts to estimate pharmaceutical loads to freshwater (Alder et al., 2010; ter Laak et al., 2010; Winker et al., 2008) and to monitor concentrations of pharmaceuticals in wastewater effluents and in streams (Hirsch et al., 1999; Kasprzyk-Hordern et al., 2008; Ternes, 1998). None of the previous studies took a perspective as we undertake in the current study, translating the loads of both human and veterinary pharmaceuticals to water into a GWF and putting this GWF in the context of the limited assimilation capacity of freshwater systems. The objective is to gain insight in the GWF of pharmaceuticals from different sources and explore which of the selected substances and emission pathways are most influential and what parameters are most important. The GWF is quantified and spatially mapped, distinguishing between GWFs related to households and hospitals and different types of livestock farming. GWFs are thereby expressed as polluted water volumes per area, but also per community, per person, and per unit of animal product (meat, milk, egg). The results are compared with the GWF of other pollutants and thus add a useful extension to the assessment of the overall WF of human society.

We consider the GWF of pharmaceuticals at three spatial levels. In a global analysis for two substances we obtain a global picture of the severity of water pollution through pharmaceuticals. At national level we estimate and contrast pharmaceutical pollution in two countries (Germany and the Netherlands) for a range of substances and compare the GWF per person related to direct pharmaceutical use to the GWF per person from the consumption of animal products (in the supply chain of which veterinary pharmaceuticals were used). In a detailed, high-resolution analysis at river basin level (for the Vecht catchment shared by Germany and the Netherlands) we identify local hotspots of water pollution through pharmaceuticals. We estimate the potential effect of the GWF per sub-catchment by calculating the water pollution level (WPL) as the ratio of the GWF to catchment runoff.

## 2 Methods and data

### 2.1 Geographical levels of analysis

At global level, where data on pharmaceutical use are extremely limited, we estimate the pollution from human use of carbamazepine and ciprofloxacin, using data on loads emitted to the aquatic environment from Oldenkamp et al. (2019). At national level, environmental loads and related GWFs are estimated for Germany (GE) and the Netherlands (NL), both for human and veterinary pharmaceuticals. Regarding human pharmaceuticals, substances from several therapeutic groups are included whereas the selection of veterinary pharmaceuticals is limited to antibiotics. Details on the substance selection are included in the Supplementary Information (SI). The basin level study for the Vecht catchment (VC) considers the same substances as on the national level. A detailed description of the catchment is in the.SI.

### 2.2 Grey water footprint and water pollution level

Water footprint (WF) assessment is a method to quantify consumptive as well as degradative freshwater use. The consumptive WF refers to the consumption of rainwater (green WF) and groundwater or surface water (blue WF). The degradative WF, called the grey WF (Hoekstra et al., 2011), refers to the volume of water that is required to assimilate pollutants, which is the volume of water needed to dilute pollutants to the extent that the quality of the ambient water remains above water quality standards (Franke et al., 2013). The GWF [m^3^ yr^−1^] is defined as the load of pollutant *L* [kg yr^−1^] divided by the difference between the maximum allowed concentration *C*_max_ [kg m^−3^] and the natural background concentration *C*_*na*t_ [kg m^−3^] (Hoekstra et al., 2011). For pharmaceuticals considered here, *C*_*na*t_ is zero. The GWF is estimated separately for different substances. The overall resultant GWF is equal to the largest GWF across the examined contaminants (Hoekstra et al., 2011). We estimate GWFs on a temporal scale of one year.

The water pollution level (WPL) in a basin or sub-catchment is defined as the ratio of the GWF [m^3^ yr^−1^] to the catchment’s runoff R [m^3^ yr^−1^] (Hoekstra et al., 2011). WPL>1 indicates that ambient water quality standards are violated. WPL is estimated in the Vecht case study on annual basis per sub-catchment. Runoff (precipitation minus evaporation) is estimated from data at a resolution of 1 km^2^ for the reference period 1961–1990 (BFG, 2019) and extrapolated to the Dutch part of the catchment as climatic and hydrological conditions are comparable.

### 2.3 Human pharmaceutical loads

Following modelling approaches presented by e.g. Alder et al. (2010) and ter Laak et al. (2010), pharmaceutical loads entering the aquatic environment as point sources are estimated as:

(1) $$L_{h}=S\times f_{e}\times \left(1-f_{r}\right)$$

where *L*_*h*_ [kg yr^−1^] is the load of a specific human pharmaceutical to water, *S* [kg yr^−1^] the sales of the pharmaceutical in a defined geographical area, *f*_*e*_ [−] the excreted fraction, and *f*_*r*_ [−] the fraction removed by wastewater treatment.

Pharmacy sales are obtained on national and VC level for GE and NL. Data on pharmaceutical use in hospitals is collected from hospital pharmacies in the VC. Substance-specific input values for excreted pharmaceutical fractions and removed fractions in wastewater treatment plants are retrieved from scientific literature (SI).

### 2.4 Veterinary pharmaceutical loads

Veterinary pharmaceutical loads are estimated separately for beef cattle, dairy cattle, pigs, broiler and laying hens, for GE and NL as a whole and for the VC. The main emission pathways via direct (excretion of grazing animals) and indirect (manure collection and application) emissions were considered (Boxall, 2008). Aggregated loads per pharmaceutical and livestock type are defined as:

(2) $$L_{t}\left[i\right]=L_{d}\left[i\right]+\sum_{m}L_{in}\left[i,m\right]$$

where *L*_*t*_[*i*] [kg yr^−1^] is the total load of a specific veterinary pharmaceutical from livestock type *i*, *L*_*d*_[*i*] [kg yr^−1^] the load from manure directly emitted to pasture land, and *L*_*in*_[*i,m*] [kg yr^−1^] the indirect load from manure type *m* (liquid or solid) applied to fields after temporary storage. Following descriptions by Boxall et al. (2004), direct loads are estimated as:

(3) $$L_{d}\left[i\right]=365\times a\left[i\right]\times f_{e}\times f_{d}\left[i\right]$$

where *a* [kg day^−1^] is the administered substance per day, *f*_*e*_ [−] the excreted fraction, and *f*_*d*_ [−] the fraction directly emitted to pasture land.

The pharmaceutical load from manure that has been stored before application to fields is estimated per livestock type *i* and manure type *m* (liquid or solid) using a first-order degradation model (Ray et al., 2017; Wang and Yates, 2008), assuming constant production of manure over time (see derivation in SI):

(4) $$L_{in}\left[i,m\right]=\frac{365}{T\left[i,m\right]}\times \left(\frac{a\left[i\right]\times f_{e}\times \left(1-f_{d}\left[i\right]\right)\times f_{man}\left[i,m\right]}{k\left[i,m\right]}\times \left(1-e^{-k\left[i,m\right]\times T\left[i,m\right]}\right)\right)$$

where (1-*f*_*d*_) [−] is the fraction of the daily production that is stored, *f*_*man*_ the fraction of manure type *m*, *k* [day^−1^] the degradation rate, *T* [days] the duration of one storage period, and 365/*T* [−] the number of storage periods per year. By definition, *k* equals ln(2) divided by the half-life of the substance (which differs per type of manure and livestock type).

Amounts of administered substances (separately for beef cattle, dairy cattle, pigs, broilers and laying hens) are estimated based on veterinary pharmaceutical sales data for GE and NL. By lack of livestock-specific data, we assume the same excretion fractions as in human metabolism. Data on pharmaceutical degradation during manure storage are obtained from literature. Data sources and assumptions are provided in the SI.

Pharmaceutical transport to water through leaching and runoff has been addressed through experimental trials (Hamscher et al., 2005; Kay et al., 2005; Ostermann et al., 2013; Pan and Chu, 2017; Popova et al., 2013; Spielmeyer et al., 2017; Stoob et al., 2007), modelling attempts (Bailey, 2015; Knäbel et al., 2016; Mackay et al., 2005) and risk assessment methods (CVPM, 2018; Menz et al., 2015), but a comprehensive method applicable for the scope of this study is lacking. Given lack of data on decay in the soil and because pharmaceuticals from agricultural use have been found in freshwater resources under agricultural fields in GE and NL (Karfusehr et al., 2018; Kivits et al., 2018), we follow here the precautionary principle by assuming that all loads applied to the field could potentially end up in freshwater. This may overestimate water quality impacts given potential degradation or accumulation in the soil (Hannappel et al., 2014; Kümmerer, 2008a). There is great variance in mobility among different pharmaceuticals (Boxall, 2008). Plant uptake or photodegradation can occur after pharmaceuticals have been applied to the field (Jechalke et al., 2014). We address this issue of potential overestimation in a sensitivity analysis presented in the SI.

### 2.5 Limit concentrations of pharmaceuticals

Although the EU included pharmaceuticals in the priority substances and watch list under the water framework directive (European Comission, 2016, 2019), there are no legally binding environmental limit concentrations for pharmaceuticals (Barbosa et al., 2016). Here, we take predicted no-effect concentrations (PNEC) as maximum allowed concentrations for the GWF calculations (Bergmann et al., 2011). PNEC values for the substances considered are taken from literature (see SI) and reach from 0.00001 μg L^−1^ for ethinylestradiol to 60 μg L^−1^ for metformin (Bergmann et al., 2011). For amantadine, for which no PNEC value is available, we have taken 0.1 μg L^−1^, the threshold value for environmental risk assessment of pharmaceuticals suggested by the European Medicines Agency (CVPM, 2018).

## 3 Results

### 3.1 Global perspective

Based on global loads for carbamazepine and ciprofloxacin to freshwater from Oldenkamp et al. (2019) and PNEC values used in this study, global GWFs of 50 billion m^3^ yr^−1^ (carbamazepine) and 14,556 billion m^3^ yr^−1^ (ciprofloxacin) were determined, which on a per capita basis is 7 m^3^ yr^−1^ (carbamazepine) and 1900 m^3^ yr^−1^ (ciprofloxacin). While the latter in particular is very substantial – compared for instance with a global nitrogen-related GWF of 1940 m^3^ yr^−1^ (Mekonnen and Hoekstra, 2015) – water pollution from pharmaceuticals is likely to increase. The IMS Institute for Healthcare Informatics (2015) predicts a global pharmaceutical consumption increase of 32% from 2015 to 2020. Klein et al. (2018) predict a global increase of human antibiotic use by 15% towards 2030 under unchanged antibiotic use policies and antibiotic consumption rates. Extrapolating the growth of global antibiotic consumption as observed in the past years, they estimate per capita consumption to rise by 161% and total consumption by 202%. This growth largely results from emerging markets, where populations and per capita consumption rise (IMS Institute for Healthcare Informatics, 2015). Growth in per capita consumption of pharmaceuticals appears to correlate to growth in gross domestic product per capita (Klein et al., 2018). For global veterinary antibiotic consumption an increase of 67% from 2010 to 2030 is predicted due to the global rise in animal product consumption and the shift towards more intensive farming practices. This trends would also lead to an increase of global GWF of pharmaceuticals.

### 3.2 National GWFs for Germany and the Netherlands

#### 3.2.1 Human pharmaceuticals

The largest GWFs of human pharmaceuticals in GE and NL, based on pharmacy sales, excretion rates and removal rates in wastewater treatment, are estimated at 190 and 193 billion m^3^ yr^−1^, respectively, resulting from the hormone ethinylestradiol, which is not used in relatively large amounts but has a comparatively low PNEC. Total GWFs for all substances are given in the SI. Although the German population is around five times larger than the Dutch population, the total Dutch GWF is larger for two out of the eleven investigated substances, namely ethinylestradiol and oxazepam.

**Fig. 2:**
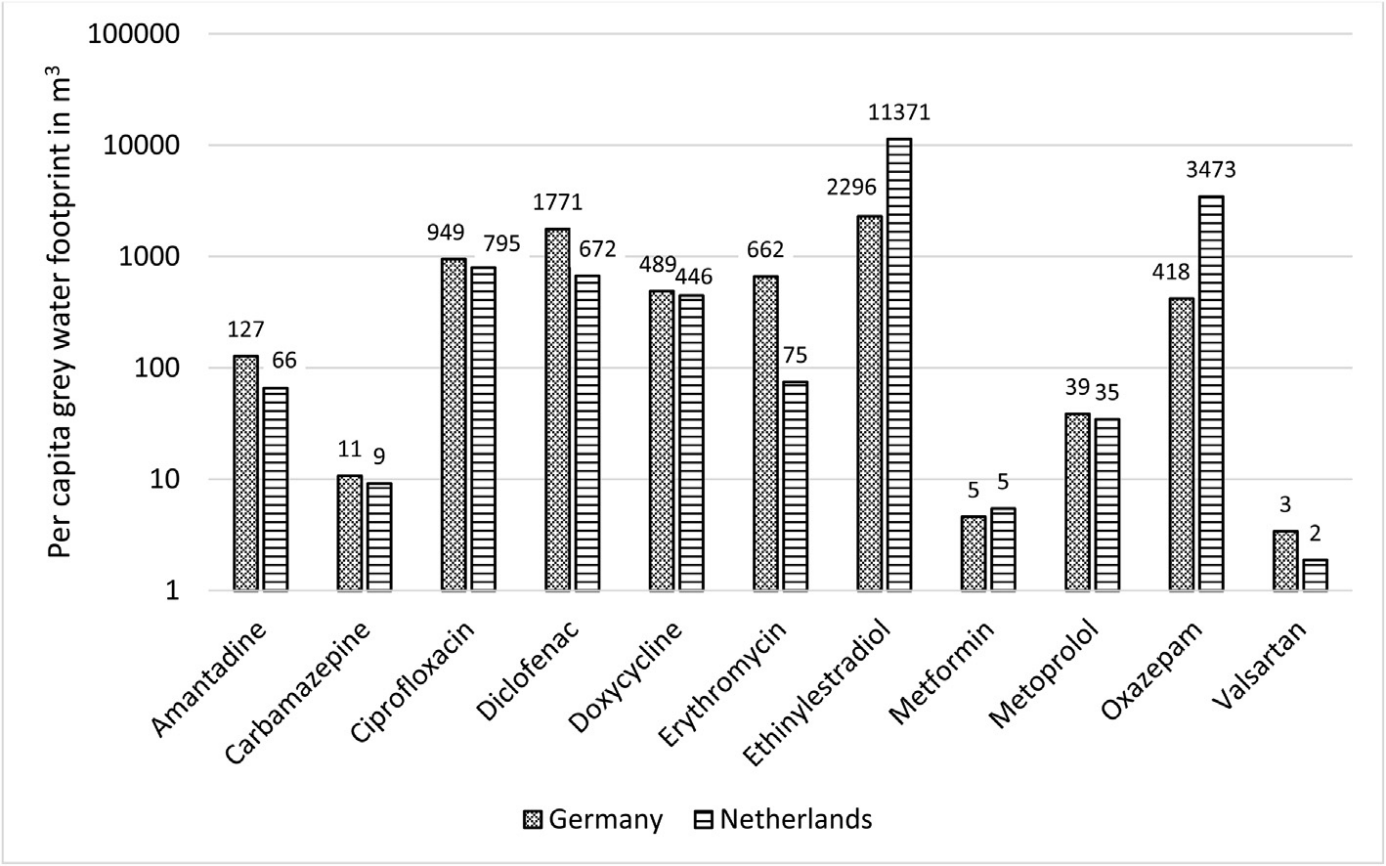
German and Dutch per capita GWFs related to human pharmaceutical use for selected substances.

Fig. 2 shows the per capita GWFs for selected substances for GE and NL. Differences in GWF are up to four orders of magnitude among compounds, resulting from a combination of different consumption volumes, excreted fractions, removed fractions in WWTPs and given PNECs. For some substances there is a one order-of-magnitude difference in per capita GWF between GE and NL. This results from different per capita consumption in the two countries, i.e. the per capita GWF linearly depends on the per capita consumption. The per capita GWF for ethinylestradiol is about five times higher for NL than for GE. GE has a larger per capita GWF than NL for eight out of the eleven substances, resulting from differing per capita sales. The largest difference in per capita GWF between the two countries is found for the antibiotic erythromycin, with a nine times larger value for GE. The national average per capita GWFs of carbamazepine for GE and NL are within the same order of magnitude as the global average (7 m^3^ yr^−1^), although slightly higher. For ciprofloxacin, the German and Dutch per capita GWFs are approximately half of the global average (1900 m^3^ yr^−1^).

#### 3.2.2 Veterinary pharmaceuticals

Between 1% and 33% of the pharmaceuticals sold to the livestock sector in Germany and the Netherlands are estimated to reach freshwater resources (Table 1), considering country specific input data. Note that the livestock sector in one country mainly contributes to water pollution in the same country, but due to cross-border manure trade, exported fractions can end up in neighbouring countries. The net trade of manure between NL and GE is from the former to the latter (Leenstra et al., 2014), so some of the pharmaceuticals from the Dutch livestock sector end up in water bodies in Germany. On the other hand, a substantial part of the water pollution in Germany flows downstream to the Netherlands. For national GWF estimations of this study, we investigated GWFs related to production of animal products and therefore present the GWF of livestock production per country, even though pollution might take place elsewhere. For the VC we evaluate manure export from the region and present this as part of the results.

**Table 1:** Estimated fractions of sold veterinary pharmaceuticals in Germany and the Netherlands that enter freshwater resources.

	Amoxicillin	Doxycycline	Oxytetracycline	Sulfamethazine	Tetracycline
Germany	9.7%	14.3%	33.2%	1.3%	24.0%
Netherlands	1.7%	5.1%	11.8%	0.9%	9.2%

The largest GWFs of livestock production are from amoxicillin and amount to 1.5 and 0.3 trillion m^3^ yr^−1^ for GE and NL, respectively, exceeding the GWFs of human pharmaceuticals. The SI provides GWFs per livestock sector and country for all substances. In GE, beef cattle contribute most (53%) to the overall GWF, whereas in NL dairy cattle contribute most (60%). Note that this distribution is a first approximation, resulting from the assumptions taken in this study regarding the distribution of pharmaceuticals over the different animal types.

Given their weight, a beef or dairy cow has a larger annual GWF than a pig or chicken (see SI). More informative, Table 2 shows the GWF per unit of animal product. Among the three meat types, beef has the largest GWF in GE (654 m^3^ kg^−1^) whereas pork has the largest GWF in NL (212 m^3^ kg^−1^). Chicken meat has the smallest GWF in both countries. Except for pork, GWFs for all products are larger in GE than in NL. As shown in the table, the pharmaceutical-related GWFs add substantially to the total WFs of the animal products as estimated previously while excluding pharmaceutical-related GWFs (Mekonnen and Hoekstra, 2012).

**Table 2:** GWFs related to selected substances per unit of animal product produced in Germany (GE) and the Netherlands (NL) compared to the total (global average) water footprint (WF) of the same products estimated earlier when excluding the GWF from veterinary pharmaceutical use.

Animal product	Unit	Grey water footprint related to veterinary pharmaceutical use										Total WF a
		Amoxicillin		Doxycycline		Oxytetracycline		Sulfamethazine		Tetracycline		
		GE	NL	GE	NL	GE	NL	GE	NL	GE	NL	
Beef meat	m^3^ kg^−1^	654	148	114	50	0.68	0.29	0.16	0.13	15	8	15
Milk	m^3^ L^−1^	15	11	3	4	0.02	0.02	0.003	0.01	0.35	0.55	1
Pig meat	m^3^ kg^−1^	51	212	8	88	0.07	0.79	0.004	0.06	2	21	6
Chicken meat	m^3^ kg^−1^	15	0.14	4	0.09	0.03	0.0006	0.002	0.0001	1	0.03	4
Egg	m^3^ kg^−1^	2	0.5	0.6	0.3	0.006	0.003	0.0003	0.0003	0.5	0.2	3

a Excluding pharmaceutical-related GWF, source: Mekonnen and Hoekstra (2012).

**Table 3:** Grey water footprint of selected substances in the German and Dutch parts of the Vecht catchment.

Substance	Grey water footprint [10^6^ m^3^ yr^−1^]		
	Households	Hospitals	Total
Amantadine	130	1.2	131
Carbamazepine	18	0.02	18
Ciprofloxacin	1465	155	1620
Diclofenac	1443	22	1465
Doxycycline	861	7	868
Erythromycin	408	15	423
Ethinylestradiol	16,104	n.d.	16,104
Metformin	9	0.03	9
Metoprolol	67	0.44	67
Oxazepam	5099	64	5163
Valsartan	4	0.02	4

#### 3.2.3 The direct and indirect pharmaceutical-related GWF of a consumer

**Fig. 3:**
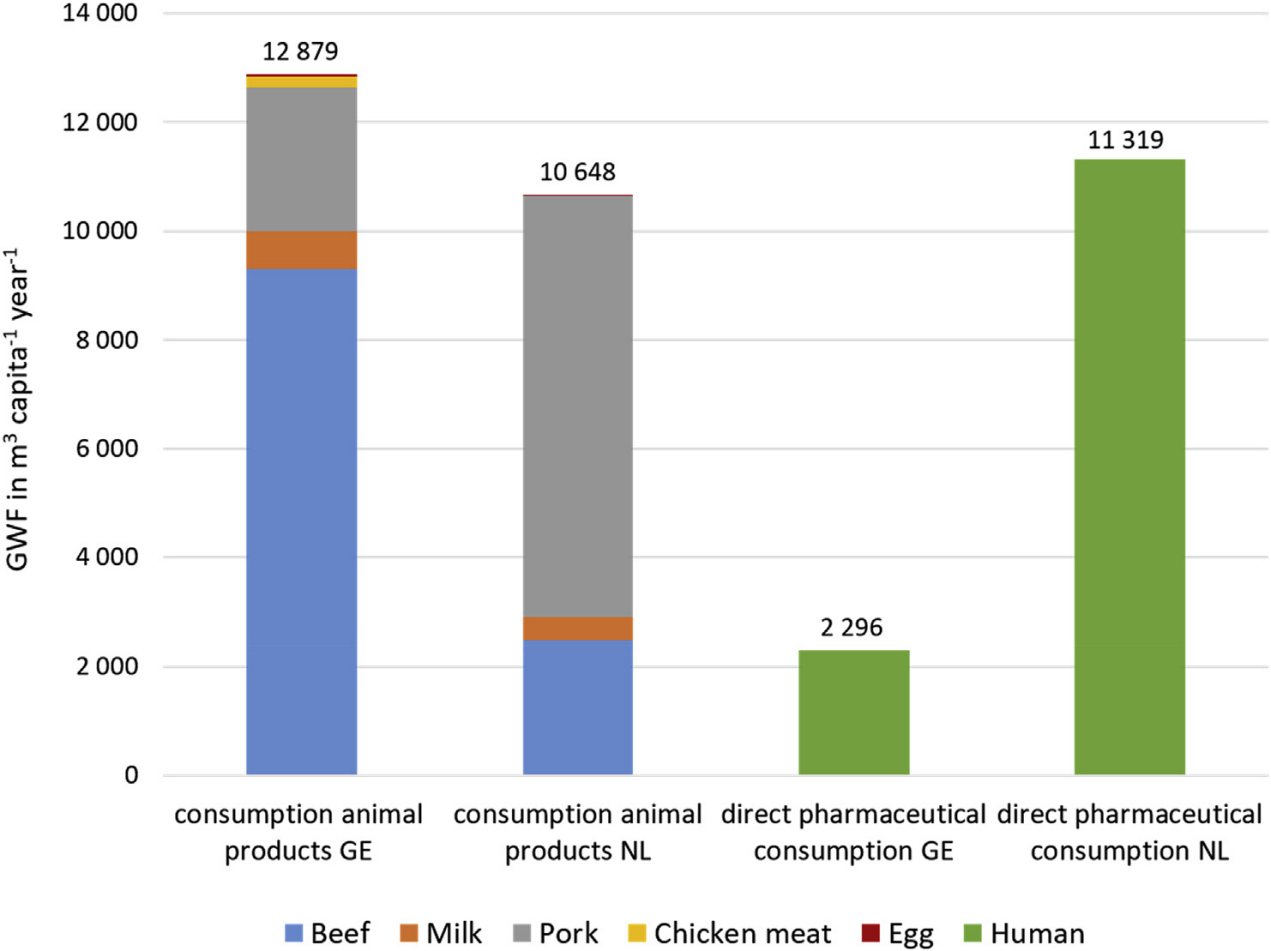
Annual GWF per capita resulting from animal product consumption (amoxicillin) and from direct pharmaceutical consumption (ethinylestradiol) in Germany (GE) and the Netherlands (NL).

For both Germany and the Netherlands, Fig. 3 shows the pharmaceutical-related GWF (for the critical substance amoxicillin) per consumer resulting from meat, milk and egg consumption next to the GWF of a consumer because of direct (human) pharmaceutical use (for the critical substance ethinylestradiol). The GWFs related to animal products in each country are based on consumption data per country and estimates on GWF per unit of product from Table 2. Pharmaceutical-related GWFs from direct human medicine use as well as through the consumption of animal products exceed earlier estimates of total consumer WFs that did not yet account for pharmaceutical pollution. Mekonnen and Hoekstra (2011) estimated the total German and Dutch WFs to be 1426 and 1466 m^3^ cap^−1^ yr^−1^, respectively, which included water consumption related to home water use and consumption of agricultural and industrial products and water pollution through nitrogen from various sources.

### 3.3 The Vecht river catchment

#### 3.3.1 Human pharmaceuticals

The VC is mapped and described in detail in the SI. Table 3 presents the GWF per substance in the VC from households and hospitals. For the most critical substance ethinylestradiol, where the GWF exclusively results from households, approximately 95% of the total GWF in the catchment results from the Dutch part. This is due to the combination of more inhabitants in the Dutch area and a higher per capita use of the substance. GWFs for all substances are presented in the SI.

There are approximately 4400 hospital beds in the region, divided over 15 hospitals (seven in GE, eight in NL). For six out of the 10 substances investigated, the GWF from hospitals adds less than 1% to the GWF from households. For ciprofloxacin, however, the GWF from hospitals amounts to about 10% of the total. The contribution of hospitals to the total GWF in the catchment is thus substance-specific.

**Fig. 4:**
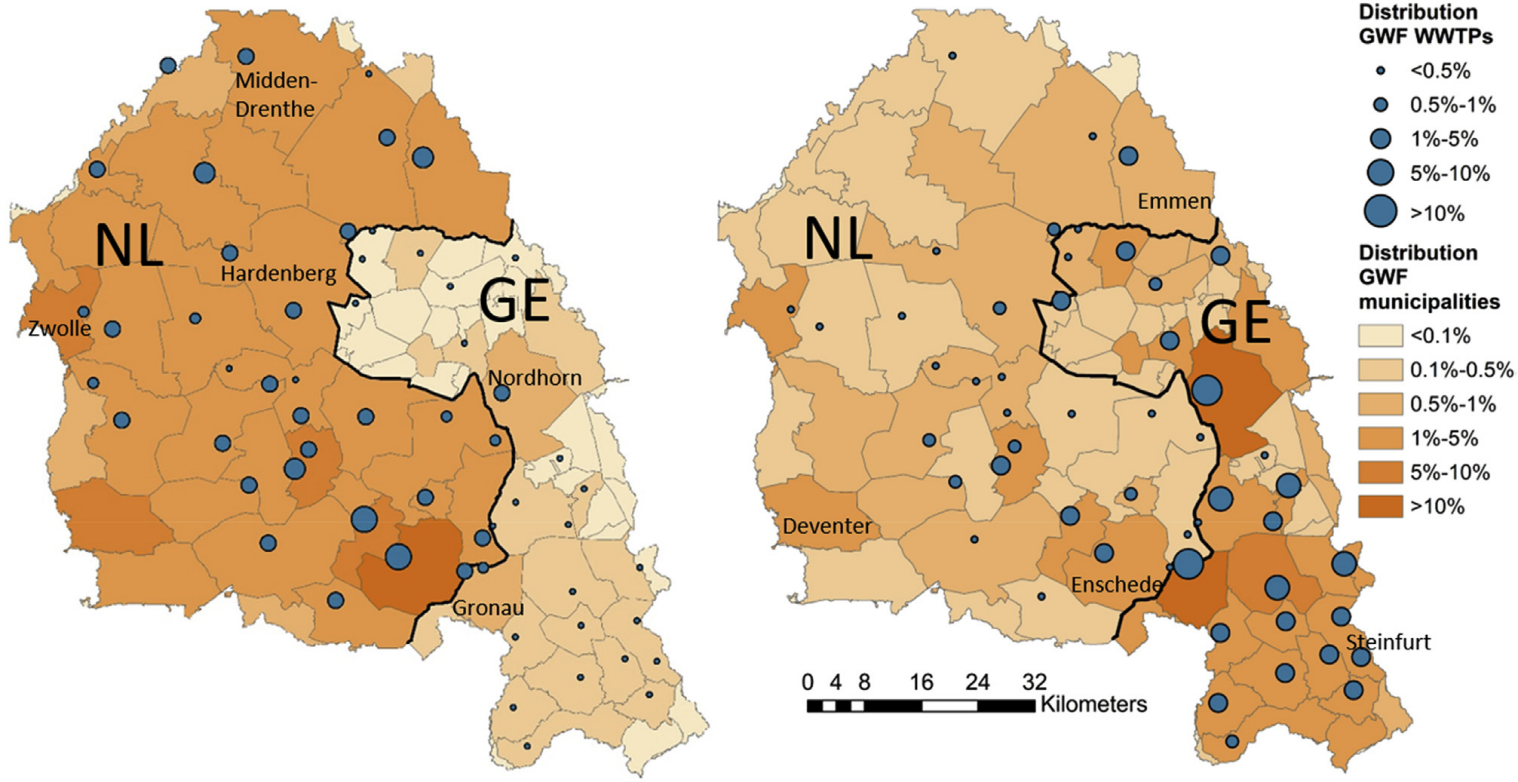
Relative contributions of municipalities and wastewater treatment plants (WWTPs) to the total GWF related to ethinylestradiol (left) and erythromycin (right) the Vecht catchment.

Fig. 4 shows the relative contributions of municipalities to the total GWF in the catchment, for ethinylestradiol (the most critical substance) and erythromycin (with quite a divergent spatial pattern). The actual loads occur through the wastewater treatment plants (WWTPs) in the catchment, the contributions of which to the total are shown as well. Though the municipalities vary in size, it can be observed that GWF hotpots occur in the more densely populated communities such as Enschede and Nordhorn. The WWTPs that contribute most to the overall GWF are obviously located in or near the municipalities that contribute most. The share of different municipalities in the overall GWF in the catchment differs across substances, explained by diverging per capita sales.

#### 3.3.2 Veterinary pharmaceuticals

The largest GWF (93 billion m^3^ yr^−1^) resulting from livestock in the VC is for amoxicillin, with the German part of the catchment contributing 53%. Whereas amoxicillin is the critical substance at catchment level, the maximum GWF on the Dutch side is determined by doxycycline. Substance-specific pharmaceutical loads and GWFs are presented in the SI.

**Fig. 5:**
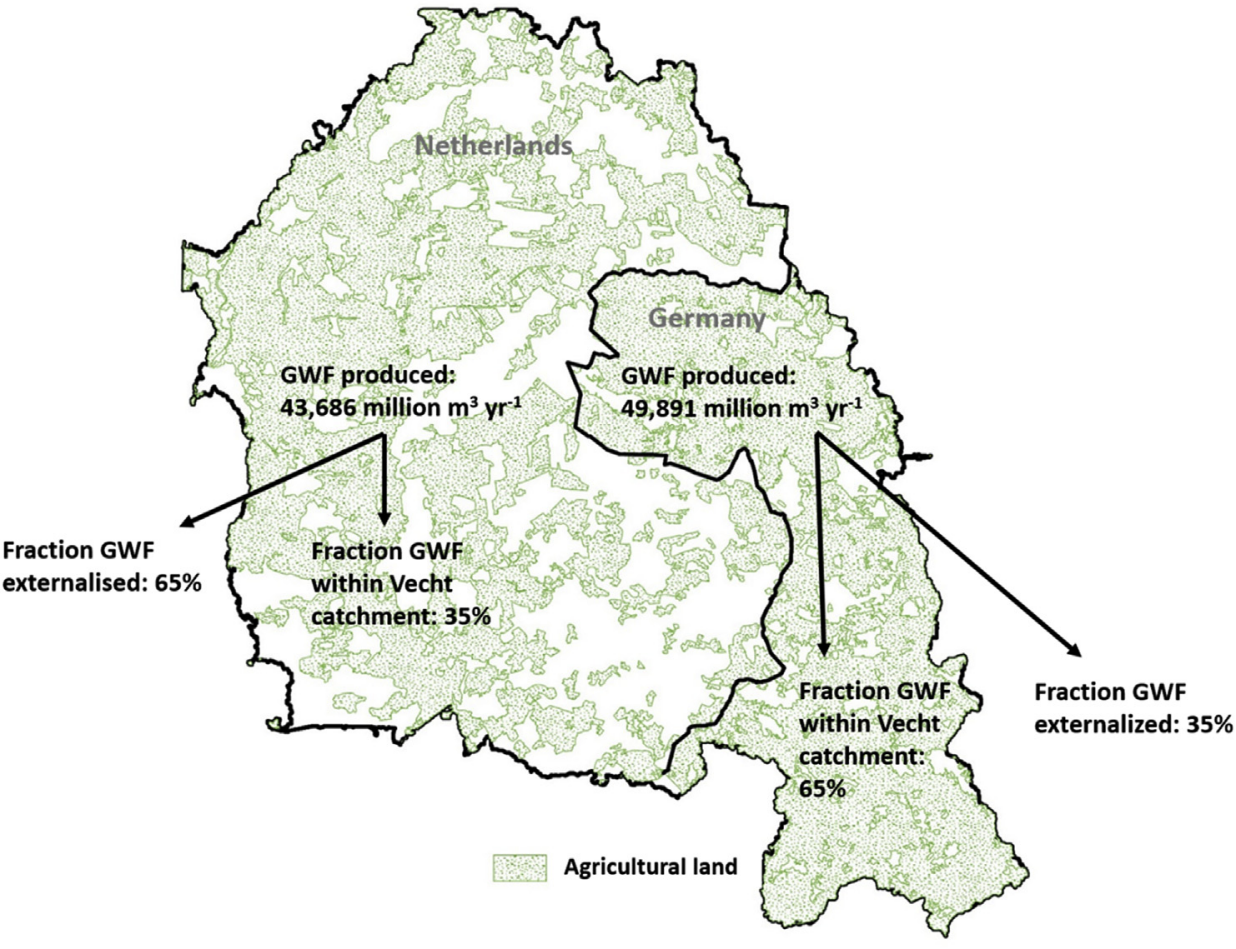
GWF related to veterinary use of amoxicillin produced in the Vecht catchment and fractions remaining within and being exported from the catchment.

Substantial amounts of manure produced in the VC are exported from the region, thus externalizing pharmaceutical emissions (Fig. 5). In the German part of the catchment, 80% is used as agricultural land (arable and grassland); in the Dutch part this is 52%. Considering a nitrate limit for manure of 170 kg N ha^−1^ yr^−1^ (European Commission, 2010) and the available agricultural land in the VC, we find a maximum possible application of approximately 25 and 38 million kg N yr^−1^ in the German and Dutch parts of the catchment, respectively. Produced nitrogen from animal excretion was estimated at 38 and 109 million kg yr^−1^ in the German and Dutch parts, respectively, taking into account livestock densities and animal-specific nitrogen excretion factors, implying manure surpluses in both parts of the catchment. The German area thus externalizes 35% of its GWF; the Dutch part even 65%.

#### 3.3.3 Water pollution levels in the Vecht catchment

**Fig. 6:**
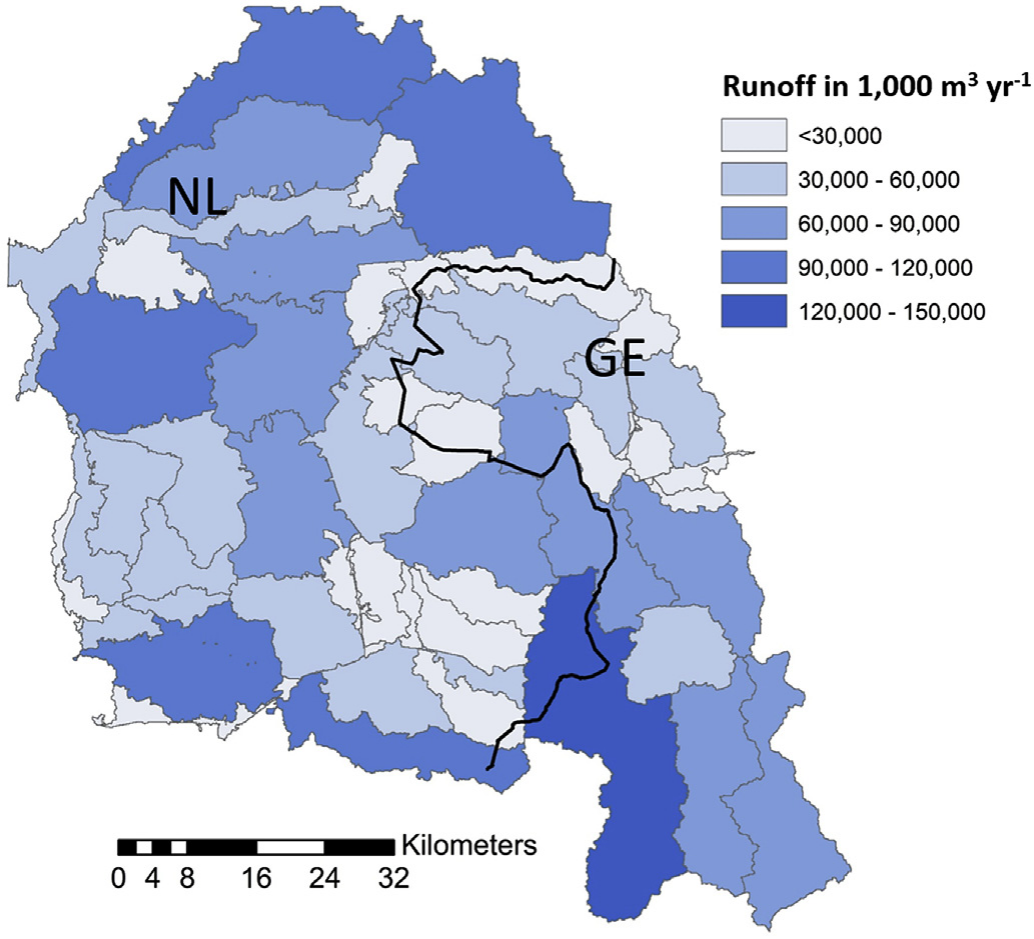
Average runoff per sub-catchment of the Vecht river catchment.

The total catchment’s runoff is about 2 billion m^3^ yr^−1^ on average. The runoff per km^2^ per sub-catchment ranges from 202,000 to 378,300 m^3^ km^−2^ and is 332,500 m^3^ km^−2^ for the catchment as a whole. Differences in runoff per sub-catchment (Fig. 6) follow from differences in hydrology and sub-catchment size.

**Fig. 7:**
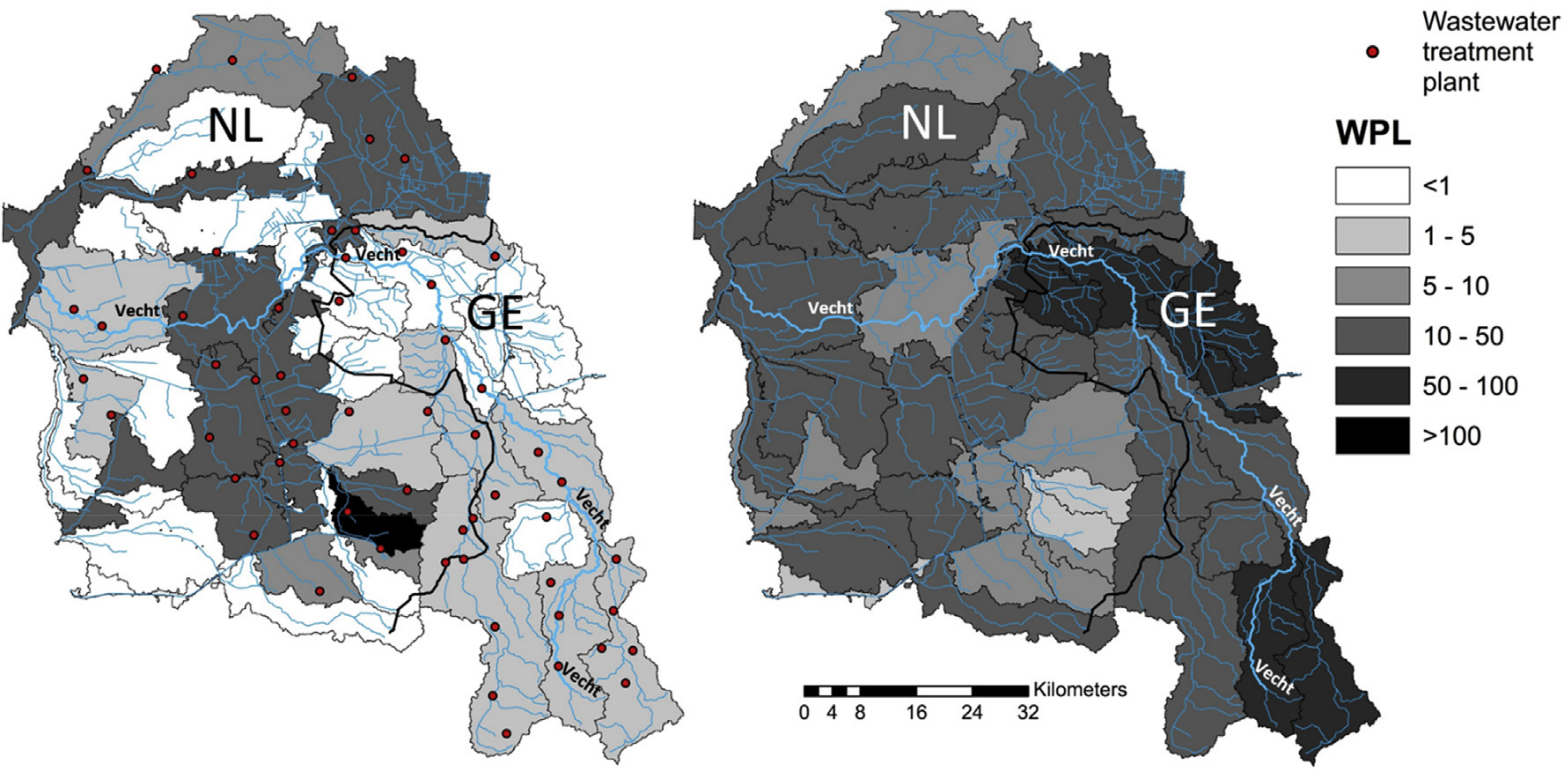
Annual average WPL in the Vecht catchment resulting from the maximum GWF of human (left) and veterinary (right) pharmaceutical use, resulting from ethinylestradiol and amoxicillin, respectively.

Fig. 7 shows the WPL in the VC resulting from ethinylestradiol, the most critical human pharmaceutical, and from amoxicillin, the most critical veterinary pharmaceutical. The total GWF related to human use of ethinylestradiol is 13 billion m^3^ yr^−1^. The total GWF related to veterinary use of amoxicillin is 48 billion m^3^ yr^−1^. For both, human and veterinary pharmaceuticals, the GWF exceeds the available runoff. WPLs across sub-catchments differ, indicating hotspots. For human pharmaceuticals, WPL is high in sub-catchments with large disposals from WWTPs. In several sub-catchments WPL<1, which means that the GWF can be assimilated by the runoff generated within the area. In NL, per capita GWF for ethinylestradiol is 4.2 times larger than in GE, contributing to higher WPLs in sub-catchments on the Dutch side. In one sub-catchment, WPL exceeds 100, demonstrating a remarkable hotspot. This high value is caused by the presence of WWTPs of two majors cities, namely Enschede and Hengelo (connecting over 274,000 inhabitants), a GWF of 11,123 m^3^ yr^−1^ per inhabitant and a relatively low runoff.

The WPL from veterinary pharmaceuticals exceeds 1 for all sub-catchments. It results from amoxicillin and distributes more homogeneously over the catchment than the WPL for ethinylestradiol from human use. While human pharmaceutical emissions enter as point sources at specific locations, veterinary emissions are diffuse and spread out, as manure is applied throughout the basin. The GWF per area related to the veterinary pharmaceutical emissions in the VC is 22 and 7 million m^3^ km^−2^ for the German and Dutch side, respectively. We observe that the sub-catchments with the lowest WPLs for veterinary pharmaceuticals, show a high WPL for human pharmaceuticals, because there is little agricultural land in densely populated areas. Note that WPLs presented here refer to locally generated pollution, which excludes incoming water pollution from upstream sub-catchments. We take this approach as we are not considering biogeochemical (decay) processes along the river streams.

## 4 Discussion

### 4.1 General observations

This study shows that per capita GWFs of human pharmaceuticals can hugely vary across substances and among countries. The latter can be explained by differences in consumption patterns (Klein et al., 2018; OECD, 2017) and wastewater treatment coverage (Oldenkamp et al., 2019). Global trends predict an increase in pharmaceutical use for both humans and livestock. If wastewater treatment in countries with increasing consumption does neither increase nor improve, GWFs of human pharmaceuticals will rise. The GWF from veterinary pharmaceuticals will increase with increasing consumption of animal products if there are no changes in application routines. To draw more robust conclusions about GWFs of pharmaceutical pollution on a global level, substance-specific information on consumption patterns as well as influential parameters have to be available and analysed within future research.

### 4.2 GWFs of pharmaceuticals in context

The global average per capita GWF from the human antibiotic ciprofloxacin estimated here is 1900 m^3^ yr^−1^. This is a lot when compared to the global average WF of 1385 m^3^ yr^−1^ per person estimated by Hoekstra and Mekonnen (2012), a value including all water consumption and nitrogen-related water pollution from households, industries and agriculture (but excluding water pollution from pharmaceuticals). National per capita GWFs from direct pharmaceutical consumption and from consumption of animal products for GE and NL exceed this WF. The WF of a consumer thus increases substantially when including pharmaceutical pollution.

The results of this study can be compared to estimated GWFs for other pollutants than pharmaceuticals. Mekonnen and Hoekstra (2015) estimate a global nitrogen-related GWF of 13 trillion m^3^ yr^−1^, which is the same order of magnitude as the global GWF of human use of ciprofloxacin found in this study. The global GWF from anthropogenic phosphorus loads was estimated by Mekonnen and Hoekstra (2018) at 147 trillion m^3^ yr^−1^. The GWFs related to nitrogen in GE and NL were estimated by Mekonnen and Hoekstra (2011) at 14 and 0.85 billion m^3^ yr^−1^, respectively, which in both cases is less than the GWF related to human and veterinary pharmaceuticals found in this research. Martinez-Alcala et al. (2018) estimated the GWF of human pharmaceuticals for a region in southern Spain and found a per capita GWF of 222 m^3^ yr^−1^ for carbamazepine resulting from measured concentrations in WWTP effluents, considering a PNEC of 1.2 μg L^−1^. This is a larger GWF than we found for carbamazepine on global, national and regional level within this study.

A novel part of this research is the link made between pharmaceutical water pollution and specific animal products. Meat and dairy production are major water users, contributing about one third of the global WF of humanity (Hoekstra (2020) while not yet considering pharmaceutical pollution. Mekonnen and Hoekstra (2012) estimate the average WF of beef to be about 15 m^3^ kg^−1^ (the sum of water consumption and nitrogen-related water pollution), while here we find the amoxicillin-related GWF of beef with a magnitude higher for NL and GE. This shows that the GWF related to veterinary pharmaceuticals raises an additional issue related to the consumption of animal products.

An interesting aspect of the Vecht study was the investigation of a transboundary catchment with intensive livestock agriculture. Yet, pharmaceutical pollution from households is dominant in the catchment as a whole, mainly because the VC externalizes 50% of the produced GWF resulting from veterinary pharmaceuticals. The contribution of hospitals to the GWF in the VC is minor, although specific substances can locally contribute substantially. We found significant differences in per capita GWF for the same human pharmaceuticals in GE and NL. For instance, the per capita GWF of erythromycin in the VC is 16-fold larger in GE than in NL. As GE is located upstream, it is likely that NL will be affected by German emissions.

### 4.3 Uncertainties and limitations of the study

Several input parameters considered in the GWF estimations come with uncertainty, mainly due to assumptions made to fill data gaps. We evaluate the sensitivity of the outcomes to several input parameters (see SI). The results show that especially changing several input parameters at the same time can lead to substantially lower or higher GWFs. One other critical input parameter for the GWF assessment is the PNEC used as maximum allowed concentration. PNECs are derived from ecotoxicological data. Depending on data availability, e.g. for different target species, an assessment factor is applied as a precautionary approach. Further, PNECs can be derived considering different endpoints of toxicity. Consequently, different PNECs exist in literature for individual compounds. The choice of PNEC influences GWF results. Besides that, there are no limit values regarding the toxicity of a mixture of pharmaceuticals in water, which could be a relevant aspect to address in future studies. Despite these uncertainties, the main conclusion that the GWF of certain pharmaceuticals is very large compared to other forms of water pollution remains unaffected.

Further, several aspects that potentially affect the results are not included in this study resulting in limitations. First, the study covers a limited number substances while there are thousands of different compounds on the market (Jorgensen, 2008). Second, we exclusively assessed the GWF related to pharmaceutical consumption, while the manufacturing of pharmaceuticals can come along with aquatic pollution as well (Fick et al., 2009; Larsson, 2014; Sim et al., 2011). Third, this study neglects that unconsumed pharmaceuticals could be disposed directly into the sewage system causing pollution (Barnett-Itzhaki et al., 2016; Persson et al., 2009; Vollmer, 2010). Fourth, pharmaceuticals purchases via additional routes (e.g. from abroad) is not included. Fifth, for the GWF estimation exclusively the excretion of parent compounds is considered while excreted metabolites can be likewise (or even more) ecotoxic and therefore environmentally relevant (Celiz et al., 2009; Kümmerer, 2008b). The exclusion of metabolites may lead to underestimation of GWFs. Sixth, WWTP removal rates are considered assuming all sewage water undergoes treatment. However, pharmaceuticals can enter the aquatic environment via storm water overflows before undergoing treatment during rainfall events (Kay et al., 2017). Seventh, for veterinary pharmaceuticals we estimate consumption per livestock sector, but neglected differences between treated and non-treated animals that correlate with factors such as farming practices and the health status, age and weight of the animals. Eighth, following the precautionary principle, the leaching and runoff fraction of veterinary pharmaceuticals was assumed to be 100%. For the regional analysis, WPL related to veterinary pharmaceuticals exceeded 1 in all sub-catchments, indicating violation of water quality standards. Especially for non-mobile and fast-degrading substances, the precautionary approach might be overestimating, but was considered the most appropriate approach due to insufficient knowledge and data availability.

An issue that deserves follow-up research is temporal variability. The GWF and WPL analysis cover a temporal span of one year and do not account for temporal variability of input parameters. However, several factors fluctuate in time, like pharmaceutical use depending on the season (Christoffels et al., 2016; Van Boeckel et al., 2014; Yu et al., 2013), periodic peaks of manure application in spring and autumn (Boxall, 2008) and climatic variations determining runoff.

Despite the given uncertainties and limitations, this study presents a method leading to a first estimate of GWFs related to pharmaceuticals for human and veterinary pharmaceuticals at three geographical levels. Given the lack of data, the presented findings give a unique and satisfying indication of water pollution related to pharmaceutical use, which can be improved in the future.

## 5 Conclusion

The severity of water pollution through pharmaceuticals is demonstrated by the estimated GWF related to the global human consumption of ciprofloxacin, amounting to 1900 m^3^ yr^−1^ per capita. This is more than the overall aggregated consumptive WF per person in the world (considering all water consumed at home, and in industries and agriculture), estimated in previous studies. The trend of increasing global human and veterinary pharmaceutical consumption rises the likelihood for growing global water pollution from pharmaceuticals. This study demonstrates that GWFs can vary substantially among compounds (influenced by loads and PNECs) as well as regions (influenced by loads), leading to different hotspots depending on the substance under investigation. Therefore, the inclusion of other pharmaceuticals into a global GWF assessment could potentially increase the GWF found in this study. One substance causing this could be ethinylestradiol, which shows the largest GWF within the national and regional GWF assessment of this study, but is not included in the global assessment. Among the veterinary substances, amoxicillin resulted in the largest GWF. The national veterinary GWF from livestock production exceeded the human GWF for both countries. As a precautionary approach is taken for the transport of veterinary pharmaceuticals in the soil and over land, we may overestimate GWFs. However, amoxicillin is known for its mobile behaviour, which justifies the precautionary approach chosen. Further, the study demonstrates that GWFs from livestock production are partly externalized to other regions due to manure export. From a consumption perspective, an individual’s pharmaceutical-related GWF depends on direct pharmaceutical consumption as well as consumption of animal products. An effective way for consumers to reduce pharmaceutical pollution thus includes eating less meat, eggs and dairy, which comes along with a large range of other environmental and health benefits as well (Willett et al., 2019). In the VC, WPLs exceed acceptable levels for both human and veterinary pharmaceutical, illustrating the severity of freshwater pollution on river basin scale. Additionally, it can be assumed that downstream catchments are receivers of runoff as well as human and veterinary pharmaceuticals from upstream through natural flow.

## CRediT authorship contribution statement

**Lara Wöhler:** Data curation, Writing - original draft. **Gunnar Niebaum:** Data curation, Writing - original draft. **Maarten Krol:** Writing - original draft. **Arjen Y. Hoekstra:** Writing - original draft.

## Declaration of competing interest

The authors declare that they have no known competing financial interests or personal relationships that could have appeared to influence the work reported in this paper.
