# Supplementary material for: The grey water footprint of human and veterinary pharmaceuticals
Source: Water Res X. 2020 Jan 16;7:100044. doi: 10.1016/j.wroa.2020.100044 (PMC7242788; doi:10.1016/j.wroa.2020.100044)
Supplement: Multimedia component 1 [file mmc1.docx]

# **Supplementary Information**

## 1 Methods and data

## 1.1 Substance selection

Table S1 presents the substances considered in the national studies for Germany (GE) and the Netherlands (NL) and in the study for the Vecht catchment. Regarding human pharmaceuticals, we consider a large variety of substances from different therapeutic classes for the grey water footprint (GWF) assessment on national and regional level. The substance selection was based on data availability. The selection of veterinary pharmaceuticals was limited to the therapeutic class of antibiotics. The substances under investigation represent compounds that are sold in large amounts in comparison to other veterinary antibiotics and/or have been detected in the environment within the case study regions (Karfusehr et al., 2018, Kivits et al., 2018, Veldman et al., 2018, Wallmann et al., 2018).

Table S1. Selected substances including therapeutic class and type of application

| Substance | Therapeutic class | Sector of application | | GWF assessment | |
| --- | --- | --- | --- | --- | --- |
|  |  | Human | Veterinary | Human | Veterinary |
| Amantadine | Antiviral | ✓ | 🗙 | ✓ | 🗙 |
| Amoxicillin | Antibiotic | ✓ | ✓ | 🗙 | ✓ |
| Carbamazepine | Antiepilepticum | ✓ | 🗙 | ✓ | 🗙 |
| Ciprofloxacin | Antibiotic | ✓ | 🗙 | ✓ | 🗙 |
| Diclofenac | NSAID^1^ | ✓ | 🗙 | ✓ | 🗙 |
| Doxycycline | Antibiotic | ✓ | ✓ | ✓ | ✓ |
| Erythromycin | Antibiotic | ✓ | ✓ | ✓ | 🗙 |
| Ethinylestradiol | Hormone | ✓ | 🗙 | ✓ | 🗙 |
| Metformin | Antidiabetic | ✓ | 🗙 | ✓ | 🗙 |
| Metoprolol | Beta blocker | ✓ | 🗙 | ✓ | 🗙 |
| Oxazepam | Psycholeptic | ✓ | 🗙 | ✓ | 🗙 |
| Oxytetracycline | Antibiotic | ✓ | ✓ | 🗙 | ✓ |
| Sulfamethazine | Antibiotic | ✓ | ✓ | 🗙 | ✓ |
| Tetracycline | Antibiotic | ✓ | ✓ | 🗙 | ✓ |
| Valsartan | Hypertensive | ✓ | 🗙 | ✓ | 🗙 |

^1^ Nonsteroidal anti-inflammatory drug

## 1.2 Characteristics of the Vecht river catchment

The Vecht river catchment (Figure S1) is a transboundary catchment shared by Germany (GE) and the Netherlands (NL). It is part of the larger Rhine river basin, also under the European Water Framework Directive (ICPR, 2015). In GE, the catchment stretches out over the states North Rhine-Westphalia and Lower Saxony, in NL over the provinces Overijssel and Drenthe. The catchment’s size is about 6,000 km^2^ of which 3,600 km^2^ are arable and grassland used for agriculture. The region has approximately 1.5 million inhabitants of which around 20% live in GE and 80% in NL. There are several larger sized cities in the area such as Nordhorn (53,000 inhabitants), Enschede (158,000 inhabitants), Hengelo (81,000 inhabitants), Almelo (72,000 inhabitants) and Zwolle (125,000 inhabitants). The Vecht catchment has relatively high livestock densities, with over 1.4 livestock units per hectare of utilized agricultural area (Eurostat, 2019). The Vecht river has its source near Laer (GE) and flows into the Zwarte Water which leads to the lake Ijssel (NL). Larger tributaries of the Vecht river include the Steinfurter Aa, Dinkel and Regge. There has been intensive human interference into the catchment’s hydrological system.


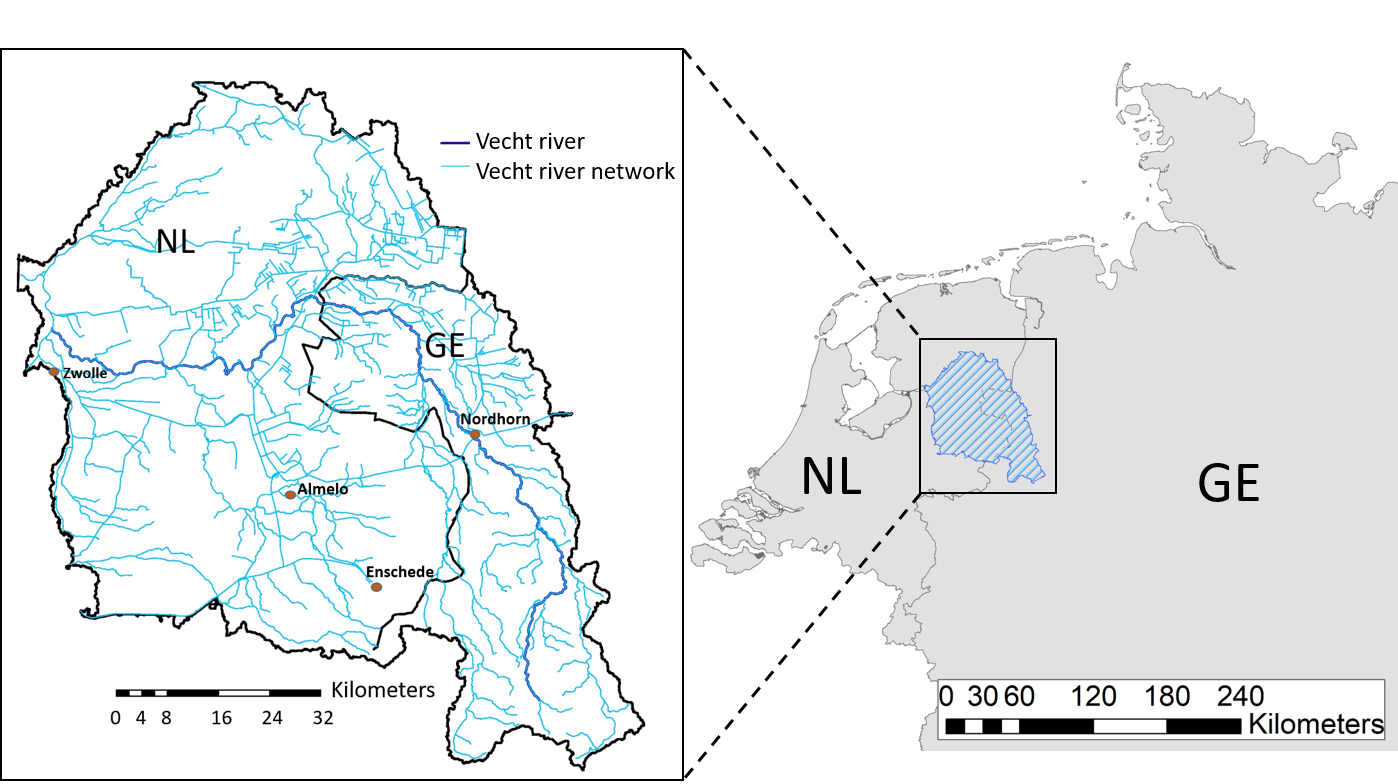


Figure S1. The Vecht river catchment, indicating the German (GE) and Dutch (NL) part of the catchment

## 1.3 Input data human pharmaceutical loads

Data on pharmaceutical sales for GE and NL were obtained from IQVIA^[[1]](#footnote-1)^ and SFK^[[2]](#footnote-2)^ respectively. Data for the Vecht catchment at the level of grouped municipalities were provided by IQVIA and SFK as well. The reference year for all pharmacy sales data is 2017. It is assumed that the amount of sold pharmaceuticals equals the amount consumed pharmaceuticals. Furthermore, local hospitals in the Vecht catchment were approached to obtain the pharmaceutical dispersion through their hospital pharmacies. For hospitals in the region that did not provide data, pharmaceutical dispersion was extrapolated from the others, differentiating between German and Dutch hospitals.

The excreted fraction from the human body varies among pharmaceuticals. We focus here on what is excreted in the same form as the parent compound. For this study, excreted fractions were determined from literature. A comprehensive list is presented in Table S2.

Table S2. As parent compound excreted fraction used for the grey water footprint estimations

| Substance | Excreted fraction [%] | Reference |
| --- | --- | --- |
| Amantadine | 86 | Moffat et al. (2011) |
| Amoxicillin | 60 | Moffat et al. (2011) |
| Carbamazepine | 6 | Kümmerer et al. (2011) |
| Ciprofloxacin | 67 | Lienert et al. (2007) |
| Diclofenac | 33.3 | Kümmerer et al. (2011) |
| Doxycycline | 40 | Moffat et al. (2011) |
| Erythromycin | 84 | Kümmerer et al. (2011) |
| Ethinylestradiol | 59 | Lienert et al. (2007) |
| Metformin | 70 | Moffat et al. (2011) |
| Metoprolol | 10 | Moffat et al. (2011) |
| Oxazepam | 10 | Moffat et al. (2011) |
| Oxytetracycline | 81 | Hirsch et al. (1999) |
| Sulfamethazine | 7.5 | Moffat et al. (2011) |
| Tetracycline | 85 | Hirsch et al. (1999) |
| Valsartan | 87 | Moffat et al. (2011) |

In GE and NL the connection rates to public wastewater treatment plants (WWTPs) are 97% and 99%, respectively (OECD, 2015). In both countries, households are responsible to treat their wastewater if they are not connected to public WWTPs. Therefore, we assume for our estimations of pharmaceutical loads that all wastewater undergoes treatment. The removal of pharmaceutical residues during treatment is dependent on substance properties and WWTP characteristics such as treatment steps, plant size and retention time (Jelić et al., 2012, Yang et al., 2011). In GE and NL, tertiary treatment (a combination of mechanical, biological treatment and nutrient removal) is predominant (OECD, 2015). Conventional treatment is not designed to specifically remove pharmaceuticals or other micropollutants (Jelić et al., 2012) and depending on the substance, fractions of pharmaceuticals are not eliminated during the treatment process. Removal fractions of pharmaceuticals were determined from literature (Table S3). We preferentially considered literature that describes pharmaceutical removal determined with flow measurements over at least 24 hours within plants with tertiary treatment and used median values for our estimations when multiple suitable data sets were identified. In case of multiple measurements for one treatment plant, average values were determined. For substances where a negative median removal was determined, we assumed that there is no elimination. For amantadine no data for removal during tertiary treatment was available. In this case, a removal fraction of 42.3% reported for biological treatment was used (Ghosh et al., 2010). No distinctions were examined for different WWTP sizes or operations. From the WWTP discharge, the wastewater is released into the aquatic environment as a point source.

Table S3. Removed pharmaceutical fractions in wastewater treatment plants selected from literature considering plants with tertiary treatment

| Substance | Removed fraction in % | References | Median removed fraction in % |
| --- | --- | --- | --- |
| Carbamazepine | -193; -6.6; 0; 0; 0; 2; 2.6; 3; 5; 9; 9.5; 10; 11; 58 | (Abbeglen and Siegrist, 2012, de Jesus Gaffney et al., 2017, Feldmann, 2005, Gurke et al., 2015, Oosterhuis et al., 2013, Radjenovic et al., 2009, Rosal et al., 2010, Sacher and Thoma, 2014, Ternes et al., 2007, Vieno et al., 2007, Zuehlke et al., 2006) | 2.8 |
| Ciprofloxacin | 57; 58; 59; 74; 79; 80.5; 82.5; 90; 92; 95.5; | (de Jesus Gaffney et al., 2017, Feldmann, 2005, Guerra et al., 2014, Lindberg et al., 2005, Mauer, 2011, Rosal et al., 2010, Vieno et al., 2007, Zorita et al., 2009) | 79.8 |
| Diclofenac | -105; -9; 0; 14; 21.8; 23; 28; 33; 37; 40; 44; | (Abbeglen and Siegrist, 2012, de Jesus Gaffney et al., 2017, Feldmann, 2005, Oosterhuis et al., 2013, Radjenovic et al., 2009, Sacher and Thoma, 2014, Ternes et al., 2007, Zorita et al., 2009) | 23 |
| Doxycycline | -174; -173; 33; 52.2 | (Lindberg et al., 2005, Rosal et al., 2010, Ternes et al., 2007) | -84 |
| Erythromycin | -2; 4.3; 14; 25; 35.4; 48.5 | (de Jesus Gaffney et al., 2017, Feldmann, 2005, Guerra et al., 2014, Radjenovic et al., 2009, Rosal et al., 2010, Ternes et al., 2007) | 19.5 |
| Ethinylestradiol | 70.5 | (Zuehlke et al., 2006) | 70.5 |
| Metformin | 96; 96; 97; 98; 99; 99.2 | (de Jesus Gaffney et al., 2017, Oosterhuis et al., 2013, Sacher and Thoma, 2014) | 97.5 |
| Metoprolol | -8.6; 2; 6.5; 16.7; 21; 21; 24.7; 25; 25; 29; 31; 65 | (de Jesus Gaffney et al., 2017, Gurke et al., 2015, Maurer et al., 2007, Oosterhuis et al., 2013, Radjenovic et al., 2009, Rosal et al., 2010, Sacher and Thoma, 2014, Ternes et al., 2007, Vieno et al., 2007, Zuehlke et al., 2006) | 22.9 |
| Oxazepam | -46; 0 | (Bijlsma et al., 2012, de Jesus Gaffney et al., 2017) | -23 |
| Valsartan | 24.4; 95 | (Gurke et al., 2015, Oosterhuis et al., 2013) | 59.7 |

## 1.4 Method and input data veterinary pharmaceutical loads

The amounts of administered substances distinguishing between livestock types are not publicly accessible, neither in GE nor in NL. For GE national substance-specific data of total antibiotic annual sales for 2017 were obtained from Wallmann et al. (2018). Data on total antibiotic sales for individual substances on postcode level (first two digits) for the German part of the Vecht catchment were provided by the German Federal Office of Consumer Protection and Food Safety (Wallmann, 2017). The reference year is 2016. For NL, the most accurate data available are the national antibiotic sales per substance group (e.g. tetracyclines). From this information, an amount sold per substance was estimated proportionally to the amounts of substance sold from each group in GE. In NL, 98% of all antibiotics are sold to the livestock sector (Van Geijlswijk et al., 2018). Since more specific data is lacking, we assume that the same applies for GE. In 2016 the cattle, pig and poultry livestock population accounted for 97% of the total livestock population in both GE and NL (Eurostat, 2019). In NL, 99% of antibiotics used in the livestock sector are given to cows, pigs and chicken^[[3]](#footnote-3)^ (Van Geijlswijk et al., 2018). As no other data are available for GE, we assume that the same ratio applies here. For the GWF estimations we differentiate between pharmaceutical loads from beef cattle^[[4]](#footnote-4)^, dairy cattle, pigs, broilers and laying hens. The overall pharmaceutical use in the livestock sector is allocated to each animal type based on their relative weight as there is no substance-specific information about the number and frequency of treatments per animal. For this reason, we take into account the number of animals per livestock type as well as the average body weight per livestock type. Livestock density data on national and regional scale were taken from CBS (2019), DESTATIS (2019b), IT.NRW (2019) and LSN (2019). Average body weights per animal type were defined based on data from CVPM (2016). No region-specific data on antibiotic usage or sales data were available for the Dutch part of the Vecht catchment. Therefore, national data were translated to regional level proportional to the livestock densities in the Vecht catchment.

Data for excreted fractions of veterinary pharmaceuticals are scattered and not comprehensively available by substance and animal type, while often not differentiating between parent compounds and metabolites (Feinman and Matheson, 1978, Halling-Sorensen et al., 2001, Kim et al., 2010a). Due to this data gap, we take excretion data from the human metabolism as the most suitable option for our estimations.

According to Montforts (1999), pigs and chicken spend the whole year inside housing. Cattle are partly outside grazing, which results in direct dung distribution on pasture land. To determine the load directly emitted to pasture land, we account for one thirds of cattle grazing for half a year (BMEL, 2018). For inside animal housing, manure is generally collected and stored before it is applied to the fields. As antibiotics are known to decay during manure storage (Dolliver et al., 2008, Song and Guo, 2014, Wang and Yates, 2008, Wang et al., 2006), this process of the excreted compounds is considered in order to determine the load emitted to fields. We assume a constant input of manure into the storage over one storage period. Even though antibiotic dissipation can depend on parameters such as temperature or manure moisture content (Wang and Yates, 2008), different studies indicate the validity of antibiotic dissipation following a first order decay as a plausible approximation (Ray et al., 2017, Wang and Yates, 2008). We therefore follow this approach for our estimations and express the constant input into storage while antibiotics are decaying as:

$$\frac{dS}{dt}=I-kS$$

where *S* [kg] is the quantity of substance in stored manure, *I* [kg day^-1^] is the constant substance input with manure input into the storage and *k* [day^-1^] is the decay constant. A schematic overview of the process is shown in Figure S2. The solution of this differential equation with starting condition S(t=0) = 0 is:

$$S\left( t \right)=\frac{I}{k}\cdot(1-e^{-k\times t})$$

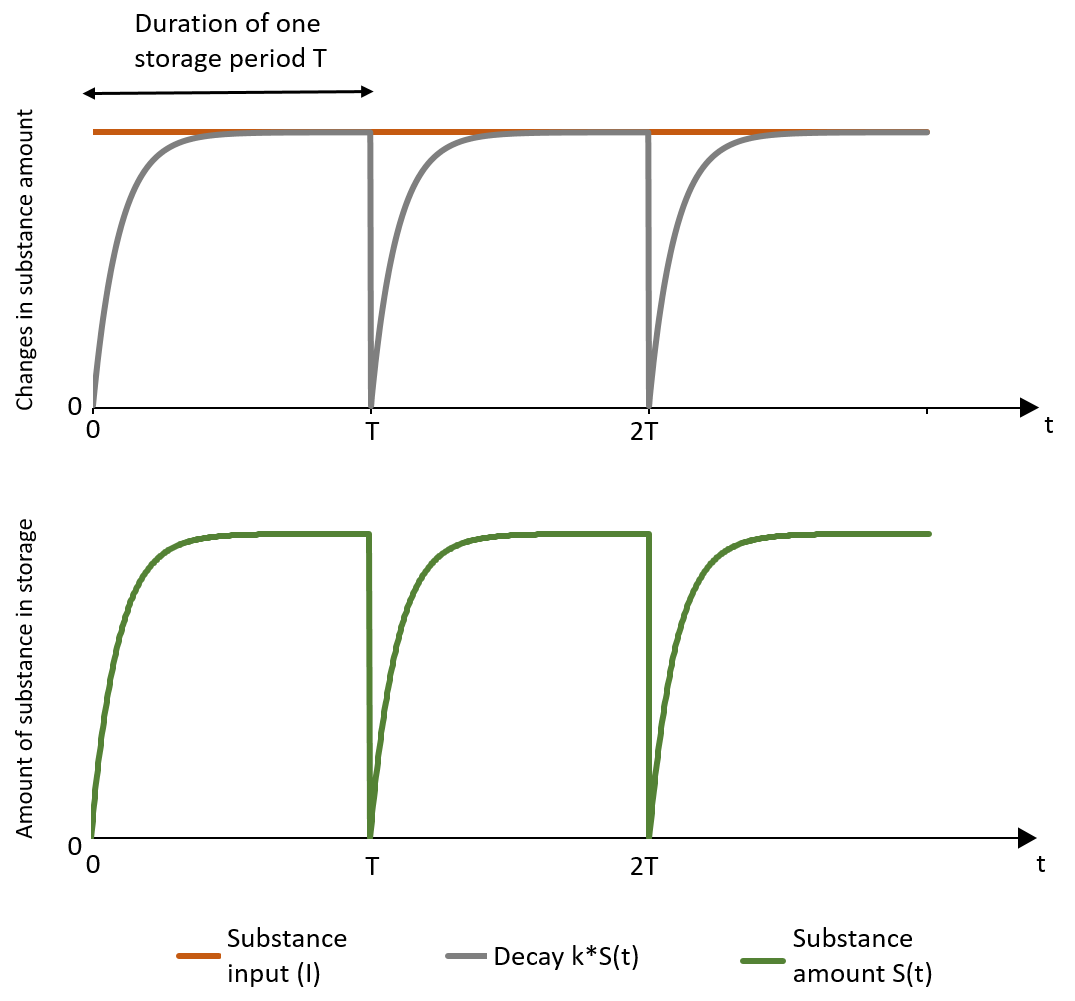


Figure S2. Schematic overview of the inflow of new pharmaceuticals in the manure storage (I) and decay of pharmaceuticals in the manure storage (kS) (upper graph) and the amount of pharmaceuticals in the manure storage (S) (lower graph). The graphs represent the example of amoxicillin (decaying with k=0.14 day^-1^) used in beef cattle and excreted in liquid manure which is stored for 75 days on average.

The average storage time of manure differs per animal type and for solid and liquid manure; data are adopted from Mackay et al. (2005). Fractions of liquid versus solid manure were obtained from DESTATIS (2019a). Substance-specific half-lives for liquid and solid manure for the different animal types were derived from Berendsen et al. (2018) and Boxall et al. (2004). In the Netherlands, 30% of chicken manure is incinerated (Leenstra et al., 2014) and therefore does not enter the environment. We consider this by assuming that the daily load from chicken excretions is reduced by 30%. This study does not account for possible degradation and transformation processes of pharmaceuticals during manure fermentation for biogas production as there is an ambiguous picture about these processes due to various influential conditions and parameters (Spielmeyer et al., 2014, UBA, 2018) and the fact that fermentation residues are generally distributed to agricultural fields after biogas production (Leenstra et al., 2014). From the loads emitted daily on pasture land and being stored over one storage period, we determine yearly loads that enter the environment.

## 1.5 PNEC values

PNEC values that are based on ecotoxicological data are used as limit concentrations for the GWF estimation. Table S4 provides an overview of applied PNEC values.

Table S4. Predicted no effect concentrations (PNEC) values selected for the grey water footprint estimations

| Substance | PNEC in µg/L | Reference |
| --- | --- | --- |
| Amoxicillin | 0.0156 | Bergmann et al. (2011) |
| Carbamazepine | 2.5 | Bergmann et al. (2011) |
| Ciprofloxacin | 0.036 | Bergmann et al. (2011) |
| Diclofenac | 0.1 | Bergmann et al. (2011) |
| Doxycycline | 0.054 | Bergmann et al. (2011) |
| Erythromycin | 0.206 | Bergmann et al. (2011) |
| Ethinylestradiol | 0.00001 | Bergmann et al. (2011) |
| Metformin | 60 | Bergmann et al. (2011) |
| Metoprolol | 3.2 | Bergmann et al. (2011) |
| Oxazepam | 0.0019 | Orias and Perrodin (2013) |
| Oxytetracycline | 1.1 | Bergmann et al. (2011) |
| Sulfamethazine | 1 | Bergmann et al. (2011) |
| Tetracycline | 0.251 | Bergmann et al. (2011) |
| Valsartan | 90 | Furtmann (2015) |

## 2 Results

## 2.1 National GWFs from human and veterinary pharmaceutical use

Table S5 presents GWFs of human pharmaceuticals for selected compounds for Germany and the Netherlands. The results are based on pharmacy sales to households and do not include dispersion through hospitals. Table S6 shows GWFs of veterinary pharmaceuticals for both countries. The results are presented per animal category and per substance. The contribution of each animal category to the overall GWF per country is displayed in Figure S3.

Table S5. Grey water footprints related to human pharmaceutical consumption in Germany and the Netherlands

| Substance | Grey water footprint [10^6^ m^3^ yr^-1^] | |
| --- | --- | --- |
|  | Germany | Netherlands |
| Amantadine | 10,537 | 1,116 |
| Carbamazepine | 888 | 156 |
| Ciprofloxacin | 75,624 | 13,014 |
| Diclofenac | 146,600 | 11,432 |
| Doxycycline | 38,472 | 7,200 |
| Erythromycin | 54,837 | 1,271 |
| Ethinylestradiol | 190,063 | 193,338 |
| Metformin | 382 | 93 |
| Metoprolol | 3,121 | 575 |
| Oxazepam | 34,625 | 59,050 |
| Valsartan | 283 | 32 |

Table S6. Grey water footprints related to veterinary pharmaceutical use in Germany and the Netherlands

| Substance | Livestock type | Grey water footprint [10^6^ m^3^ yr^-1^] | |
| --- | --- | --- | --- |
|  |  | Germany | Netherlands |
| Amoxicillin | Beef cattle | 782,335 | 63,429 |
|  | Dairy cattle | 408,787 | 152,615 |
|  | Pig | 255,777 | 40,056 |
|  | Broiler | 20,156 | 143 |
|  | Laying hen | 1,757 | 24 |
|  | Total | 1,468,812 | 256,267 |
| Doxycyclin | Beef cattle | 136,343 | 21,285 |
|  | Dairy cattle | 71,242 | 51,213 |
|  | Pig | 39,571 | 16,653 |
|  | Broiler | 4,939 | 94 |
|  | Laying hen | 544 | 20 |
|  | Total | 252,639 | 89,265 |
| Oxytetracycline | Beef cattle | 819 | 126 |
|  | Dairy cattle | 428 | 305 |
|  | Pig | 355 | 149 |
|  | Broiler | 35 | 1 |
|  | Laying hen | 5 | 0.2 |
|  | Total | 1,642 | 581 |
| Sulfamethazine | Beef cattle | 197 | 57 |
|  | Dairy cattle | 103 | 137 |
|  | Pig | 18 | 11 |
|  | Broiler | 3 | 0.1 |
|  | Laying hen | 0.3 | 0.01 |
|  | Total | 321 | 205 |
| Tetracycline | Beef cattle | 18,322 | 3,239 |
|  | Dairy cattle | 9,573 | 7,793 |
|  | Pig | 9,262 | 3,898 |
|  | Broiler | 1,497 | 29 |
|  | Laying hen | 324 | 12 |
|  | Total | 38,977 | 14,970 |


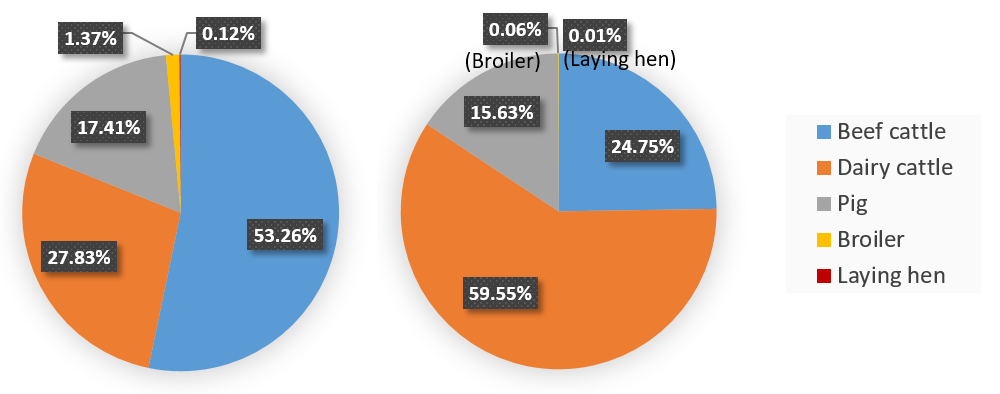


Figure S3. Relative contribution of different animal types to the overall grey water footprint of amoxicillin in Germany (left) and the Netherlands (right)

Table S7 shows the annual GWFs per animal type in Germany and the Netherlands for all substances considered. As broiler have a lifetime below one year, we do not display annual GWFs for broiler, but the GWF per lifetime. The largest average annual GWFs per animal was determined for beef and dairy cattle. Broiler showed the smallest GWF per animal, resulting also from the estimated lifetime of 37.5 days on average (Santonja et al., 2017).

Table S7. Annual GWFs per animal type for selected substances in Germany (GE) and the Netherlands (NL)

| Animal type |  | Grey water footprint | | | | | | | | | |
| --- | --- | --- | --- | --- | --- | --- | --- | --- | --- | --- | --- |
|  |  | Amoxicillin | | Doxycycline | | Oxytetracycline | | Sulfamethazine | | Tetracycline | |
|  |  | GE | NL | GE | NL | GE | NL | GE | NL | GE | NL |
| Beef cattle | m^3^ yr^-1^ | 99,683 | 52,744 | 17,373 | 17,699 | 104 | 105 | 25 | 47 | 2,334 | 2,693 |
| Dairy cattle | m^3^ yr^-1^ | 99,683 | 52,744 | 17,373 | 17,699 | 104 | 105 | 25 | 47 | 2,334 | 2,693 |
| Pig | m^3^ yr^-1^ | 9,672 | 3,230 | 1,496 | 1,343 | 13 | 12 | 1 | 1 | 350 | 314 |
| Broiler | m^3^ animal^-1^ | 22 | 0.2 | 5 | 0.2 | 0.04 | 0.001 | 0.003 | 0.0001 | 2 | 0.05 |
| Laying hen | m^3^ yr^-1^ | 42 | 0.49 | 13 | 0.41 | 0.11 | 0.003 | 0.01 | 0.0003 | 8 | 0.24 |

## 2.2 Grey water footprints of veterinary pharmaceuticals in the Vecht catchment

Table S8 presents the GWFs of human pharmaceutical consumption for the Vecht catchment as a whole and for the German and Dutch parts individually. Table S9 shows loads and GWFs per animal category for the veterinary antibiotics considered in the research.

Table S8. Grey water footprint of human pharmaceuticals for the German and the Dutch part of the Vecht catchment

| Substance | Grey water footprint (10^6^ m^3^ yr^-1^) | | |
| --- | --- | --- | --- |
|  | German part of the Vecht catchment | Dutch part of the Vecht catchment | Total Vecht catchment |
| Amantadine | 40 | 90 | 130 |
| Carbamazepine | 3 | 15 | 18 |
| Ciprofloxacin | 308 | 1,157 | 1,465 |
| Diclofenac | 537 | 904 | 1,443 |
| Doxycycline | 180 | 681 | 861 |
| Erythromycin | 318 | 90 | 408 |
| Ethinylestradiol | 798 | 15,306 | 16,104 |
| Metformin | 1 | 8 | 9 |
| Metoprolol | 11 | 56 | 67 |
| Oxazepam | 159 | 4940 | 5,099 |
| Valsartan | 0.9 | 3 | 4 |
| Inhabitants | 304,000 | 1,244,000 | 1,548,000 |

Table S9. Estimated loads and grey water footprints (GWFs) of veterinary pharmaceuticals per animal category in the Vecht catchment, distinguished between Germany (GE) and the Netherlands (NL)

| Substance | Animal type | Load GE Vecht  [kg yr^-1^] | Load NL Vecht  [kg yr^-1^] | GWF GE Vecht  [10^6^ m3 yr^-1^] | GWF NL Vecht  [10^6^ m^3^ yr^-1^] | GWF total Vecht  [10^6^ m^3^ yr^-1^] |
| --- | --- | --- | --- | --- | --- | --- |
| Amoxicillin | Beef cattle | 161 | 172 | 10,345 | 11,021 | 21,366 |
|  | Dairy cattle | 83 | 421 | 5,329 | 26,983 | 32,313 |
|  | Pig | 483 | 88 | 30,948 | 5,658 | 36,605 |
|  | Broiler | 47 | 0.33 | 3,003 | 21 | 3,024 |
|  | Laying hen | 4 | 0.05 | 267 | 4 | 270 |
|  | Total | 778 | 682 | 49,891 | 43,687 | 93,578 |
| Doxycycline | Beef cattle | 213 | 200 | 13,631 | 12,802 | 26,434 |
|  | Dairy cattle | 110 | 489 | 7,022 | 31,343 | 38,366 |
|  | Pig | 257 | 127 | 16,483 | 8,142 | 24,625 |
|  | Broiler | 40 | 0.74 | 2,533 | 48 | 2,580 |
|  | Laying hen | 4 | 0.16 | 284 | 10 | 294 |
|  | Total | 623 | 817 | 39,953 | 52,346 | 92,299 |
| Oxytetracycline | Beef cattle | 22 | 24 | 705 | 3,782 | 4,487 |
|  | Dairy cattle | 11 | 59 | 2,500 | 1,474 | 3,974 |
|  | Pig | 39 | 23 | 321 | 7 | 328 |
|  | Broiler | 5 | 0.11 | 38 | 2 | 40 |
|  | Laying hen | 0.6 | 0.03 | 4,974 | 6,804 | 11,778 |
|  | Total | 78 | 106 | 608 | 844 | 1,452 |
| Sulfamethazine | Beef cattle | n.d. | 10 | 0 | 633 | 633 |
|  | Dairy cattle | n.d. | 24 | 0 | 1,550 | 1,550 |
|  | Pig | n.d. | 1.6 | 0 | 100 | 100 |
|  | Broiler | n.d. | 0.01 | 0 | 1 | 1 |
|  | Laying hen | n.d. | 0.00 | 0 | 0 | 0 |
|  | Total | n.d. | 36 | 0 | 2,284 | 2,284 |
| Tetracycline | Beef cattle | 94 | 141 | 6,041 | 9,055 | 15,097 |
|  | Dairy cattle | 49 | 346 | 3,112 | 22,170 | 25,282 |
|  | Pig | 247 | 138 | 15,853 | 8,858 | 24,711 |
|  | Broiler | 49 | 1.1 | 3,155 | 67 | 3,222 |
|  | Laying hen | 11 | 0.44 | 695 | 28 | 723 |
|  | Total | 450 | 627 | 28,857 | 40,178 | 69,035 |

## 2.3 Sensitivity analysis for the national and river basin level

To understand the effects of input data and underlying assumptions on the results, a sensitivity analysis has been carried out for several parameters considered in the national human and veterinary GWF estimations: sales and consumption data, excretion rates, removal in WWTPs, degradation during manure storage, leaching and runoff after manure application to fields.

In the GWF analysis we assume that the amount of sold pharmaceuticals equals the consumption of pharmaceuticals. Several studies report about pharmaceutical waste resulting from medicines that were not taken by patients due to e.g. change in prescription, expired products or death of patients (Bekker, 2018, Besse and Garric, 2010, Götz and Keil, 2007, Paut Kusturica et al., 2017, Persson et al., 2009, West et al., 2014). Pharmaceutical fractions that were not taken by patients that are mentioned by Besse and Garric (2010) and Götz and Keil (2007) vary from 0% to 50%. Following this range, we chose a 25% fraction of untaken pharmaceuticals for the sensitivity analysis. The same value was chosen for the analysis of veterinary pharmaceuticals.

Uncertainty further exists regarding excreted pharmaceutical fractions as these are not only influenced by the pharmacokinetics of the substance but also by factors such as the dosage form and the age or sex of the target body (Feldmann, 2005, Mauer, 2011), which were not specifically considered within this study. To understand the effect of varying excreted fractions on the study results, we apply a 10% increase or decrease of the excreted fraction used for the GWF assessment within the sensitivity analysis. For WWTP removal efficiencies, the literature presents ranges for various substances. Per substance, the median determined from a selection of literature values was used within this study. For the sensitivity analysis, the minimum and maximum values from the literature selection were applied, also including negative removal rates. In case of single values available, a deviation of 10% was used as minimum and maximum. The sensitivity to the degradation rate of pharmaceuticals during manure storage was evaluated by assuming changes in manure storage. While we used storage times specified per animal type and manure type, Weinfurtner (2011) estimates a general storage time between 1 and 4 month depending on the vegetation period. For the sensitivity analysis we therefore assume 1 and 4 month storage time. Pharmaceutical leaching and runoff to water bodies was not modelled within this study, taking the precautionary approach of assuming that all pharmaceuticals applied to the field could end up in water. For the sensitivity analysis we consider leaching-runoff fractions below 100%. Several studies investigating the leaching of pharmaceuticals report different environmental behaviour in soil water matrices for different antibiotic groups (Hamscher et al., 2005, Kay et al., 2005, Kim et al., 2010b, Lahr et al., 2017, Ostermann et al., 2013, Pan and Chu, 2016, Pan and Chu, 2017). Even though systematic understanding of leaching behaviour of individual substances is poor, these studies jointly indicate that sulfonamides are more mobile than tetracyclines and therefore more likely to leach to groundwater. For amoxicillin Kim et al. (2012) predict high mobility. Compiling this information, we qualitatively determine a high risk of leaching for amoxicillin and sulfamethazine and a low risk of leaching for doxycycline, oxytetracycline and tetracycline. For the sensitivity analysis we express this as 95% and 5% of the initially applied mass entering water bodies for a high and low risk respectively. Overland runoff is much less than leaching. Following model results by Bailey (2015), we assume an overland runoff to water bodies of 0.15% of the initially applied load.

The effects of changing input parameters on the GWF estimations are presented in Tables S10 and S11. The sensitivity of the GWF results was assessed towards a combination of changing input parameters. Depending on the substance, human GWFs substantially respond to changes in input parameters. As no country-specific parameters were adjusted, the effect of changing input parameters on the results is identical for GE and NL. A 75% consumption of the sold pharmaceuticals, results in a 25% decrease of GWF for all substances. Changing the input parameters of excreted fraction as well as WWTP removal, effects results differently per substance. The effect of adjusting those parameters on the results considerable. Especially for the combination of larger excreted fractions and minimal WWTP removal, there is a large effect on the GWF results. For five out of the 11 substances, GWF increases by over 100% even though only 75% of the sold pharmaceuticals are assumed to be consumed. This reflects the importance of reliable data regarding these parameters for GWF estimations.

Table S10. Changes in human GWF results due to changed input parameters

|  | 75% appliance | | | | | | |
| --- | --- | --- | --- | --- | --- | --- | --- |
|  |  | -10% excreted fraction | | | +10% excreted fraction | | |
|  |  |  | min WWTP removal | max WWTP removal |  | min WWTP removal | max WWTP removal |
| Amantadine | -25% | -33% | -21% | -44% | -18% | -3% | -32% |
| Carbamazepine | -25% | -33% | +103% | -71% | -18% | +149% | -64% |
| Ciprofloxacin | -25% | -33% | +43% | -85% | -18% | +75% | -82% |
| Diclofenac | -25% | -33% | +78% | -51% | -18% | +118% | -40% |
| Doxycycline | -25% | -33% | +85% | -68% | -18% | +126% | -61% |
| Erythromycin | -25% | -33% | -14% | -57% | -18% | +5% | -47% |
| Ethinylestradiol | -25% | -33% | -16% | -49% | -18% | +10% | -45% |
| Metformin | -25% | -33% | +8% | -78% | -18% | +32% | -74% |
| Metoprolol | -25% | -33% | -5% | -69% | -18% | +16% | -63% |
| Oxazepam | -25% | -33% | -1% | not assessed^1^ | -18% | +20% | not assessed^1^ |
| Valsartan | -25% | -33% | +27% | -92% | -18% | +55% | -90% |

^1^ The maximum WWTP removal for Oxazepam was found as 0. As the median was determined negative, we selected 0 removal for the GWF assessment and did not further assess this in the sensitivity analysis.

For the sensitivity analysis of the national veterinary GWF estimations, country-specific input parameters were changed and results are presented in Table S11. Especially the excreted fraction as well as the manure storage time appear to influence the results.

Table S11: Changes in veterinary GWF results due to changed input parameters

|  | | 75% appliance | | | | | | | | | | |
| --- | --- | --- | --- | --- | --- | --- | --- | --- | --- | --- | --- | --- |
|  |  |  | -10% excreted fraction | | | | | +10% excreted fraction | | | | |
|  |  |  |  | 1 month manure storage | | 4 months manure storage | |  | 1 month manure storage | | 4 months manure storage | |
|  |  |  |  |  | runoff, leaching |  | runoff, leaching |  |  | runoff, leaching |  | Runoff, leaching |
| Germany | Amoxicillin | -25% | -29% | -21% | +35% | +43% | -38% | -30% | 26% | +34% | -42% | -34% |
|  | Doxycycline | -25% | -27% | -23% | +23% | +26% | -40% | -36% | -94% | -94% | -97% | -97% |
|  | Oxytetracycline | -25% | -27% | -23% | +20% | +24% | -39% | -36% | -94% | -94% | -97% | -97% |
|  | Sulfamethazine | -25% | -29% | -21% | +22% | +30% | -41% | -34% | +14% | +21% | -45% | -38% |
|  | Tetracycline | -25% | -27% | -23% | +39% | +43% | -38% | -34% | -93% | -93% | -97% | -97% |
|  | | | | | | | | | | | | |
| Netherlands | Amoxicillin | -25% | -30% | -20% | +14% | +25% | -35% | -25% | +7% | +17% | -40% | -30% |
|  | Doxycycline | -25% | -28% | -22% | +18% | +24% | -38% | -32% | -94% | -94% | -97% | -97% |
|  | Oxytetracycline | -25% | -28% | -22% | +17% | +22% | -37% | -32% | -94% | -94% | -97% | -97% |
|  | Sulfamethazine | -25% | -30% | -20% | +3% | +14% | -38% | -27% | -3% | +6% | -42% | -32% |
|  | Tetracycline | -25% | -29% | -21% | +28% | +35% | -37% | -30% | -94% | -93% | -97% | -97% |

## References

Abbeglen, C. & Siegrist, H. 2012. Micro pollution in communal wastewater (Mikroverunreinigungen aus kommunalem Abwasser). Bern, Switzerland: Federal Office for the Environment.

Bailey, C. 2015. The overland transport of veterinary antibiotics. PhD Thesis, Technical University Aachen, Aachen, Germany.

Bekker, C. 2018. Sustainable use of medication: Medication waste and feasibility of redispersing. PhD Thesis, University of Utrecht, Utrecht, the Netherlands.

Berendsen, B. J. A., Lahr, J., Nibbeling, C., et al. 2018. The persistence of a broad range of antibiotics during calve, pig and broiler manure storage. Chemosphere, 204**,** 267-276.

Bergmann, A., Fohrmann, R. & Weber, F.-A. 2011. Compilation ofonitoring data on environmental concentrations of pharmaceuticals (Zusammenstellung von Monitoringdaten zu Umweltkonzentrationen von Arzneimitteln). Dessau-Roßlau, Germany: German Environment Agency (UBA).

Besse, J. P. & Garric, J. 2010. Environmental risk assessment and prioritization strategies for human pharmaceuticals, review and discussion. In: Roig, B. (ed.) Pharmaceuticals in the Environment: Current knowledge and need assessment to reduce presence and impact. London: IWA Publishing.

Bijlsma, L., Emke, E., Hernandez, F., et al. 2012. Investigation of drugs of abuse and relevant metabolites in Dutch sewage water by liquid chromatography coupled to high resolution mass spectrometry. Chemosphere, 89**,** 1399-406.

BMEL 2018. Understanding Farming. Berlin, Germany: Federal Ministry of Food and Agriculture (BMEL).

Boxall, A. B., Fogg, L. A., Blackwell, P. A., et al. 2004. Veterinary medicines in the environment. Rev Environ Contam Toxicol, 180**,** 1-91.

CBS. 2019. Agriculture; animals and land use per municipality (Landbouw; gewassen, dieren en grondgebruik naar gemeente) [Online]. The Netherlands: Statistics Netherlands (CBS). Available: https://www.cbs.nl/ [Accessed 16.04.2019].

CVPM 2016. Guideline on environmental impact assessment for veterinary medicinal products in support of the VICH guidelines GL6 and GL38. London, UK: European Medicines Agency.

de Jesus Gaffney, V., Cardoso, V. V., Cardoso, E., et al. 2017. Occurrence and behaviour of pharmaceutical compounds in a Portuguese wastewater treatment plant: Removal efficiency through conventional treatment processes. Environ Sci Pollut Res Int, 24**,** 14717-14734.

DESTATIS. 2019a. Agricultural businesses that distribute manure on arable or grassland, depending on manure type 2015 (Landwirtschaftliche Betriebe, die flüssigen Wirtschaftsdünger auf Ackerland oder Dauergrünland ausgebracht haben, nach Wirtschaftsdüngerarten 2015) [Online]. Wiesbaden, Germany: Federal Statistical Office of Germany. Available: https://www-genesis.destatis.de [Accessed 20.06.2019].

DESTATIS. 2019b. Animals and animal production (Tiere und tierische Erzeugung) [Online]. Wiesbaden, Germany: Federal Statistical Office of Germany. Available: https://www-genesis.destatis.de [Accessed 20.08.2019].

Dolliver, H., Gupta, S. & Noll, S. 2008. Antibiotic degradation during manure composting. J Environ Qual, 37**,** 1245-53.

Eurostat. 2019. Eurostat Database [Online]. Available: https://ec.europa.eu/eurostat/data/database [Accessed 05.09.2019].

Feinman, S. & Matheson, J. 1978. Draft environmental impact statement subtherapeutic antibacterial agents in animal feeds. Rockville, U.S., U.S. Bureau of Veterinary Medicine Food and Drug Administration.

Feldmann, D. F. 2005. Modelling behaviour and fate of pharmaceuticals in hospital wastewater and evaluation concept of their ecotoxicological risk (Modellberechnungen zum Verhalten und Verbleib von Arzneimittelrückständen im Krankenhausabwasser und Beurteilungsmöglichkeiten ihres ökotoxikologischen Gefährdungspotentials). PhD Thesis, Technische Universität Berlin, Berlin, Germany.

Furtmann, K. 2015. ECHO-substance survey: Sartans (ECHO-Stoffbericht: Sartane (Blutdrucksenker)). Recklinghausen, Germany: State Agency for Nature, Environment and Consumer Protection of Northrhine-Westphalia (LANUV).

Ghosh, G. C., Nakada, N., Yamashita, N., et al. 2010. Occurrence and fate of oseltamivir carboxylate (Tamiflu) and amantadine in sewage treatment plants. Chemosphere, 81**,** 13-7.

Götz, K. & Keil, F. 2007. Drug disposal in private households: Does the disposal of pharmaceuticals via domestic sanitary devices contribute to water contamination? Umweltwissenschaften und Schadstoff-Forschung, 19**,** 180-188.

Guerra, P., Kim, M., Shah, A., et al. 2014. Occurrence and fate of antibiotic, analgesic/anti-inflammatory, and antifungal compounds in five wastewater treatment processes. Sci Total Environ, 473-474**,** 235-43.

Gurke, R., Rossler, M., Marx, C., et al. 2015. Occurrence and removal of frequently prescribed pharmaceuticals and corresponding metabolites in wastewater of a sewage treatment plant. Sci Total Environ, 532**,** 762-70.

Halling-Sorensen, B., Jensen, J., Tjornelund, J., et al. 2001. Worst-Case Estimations of Predicted Environmental Soil Concentrations. (PEC) of Selected Veterinary Antibiotics and Residues Used in Danish Agriculture. In: Kümmerer, K. (ed.) Pharmaceuticals in the Environment. Berlin, Heidelberg: Springer.

Hamscher, G., Pawelzick, H. T., Höper, H., et al. 2005. Different behaviour of tetracyclines and sulfonamides in sandy soils after repeated fertilization with liquid manure. Environmental Toxicology and Chemistry, 24**,** 861-868.

Hirsch, R., Ternes, T., Haberer, K., et al. 1999. Occurrence of antibiotics in the aquatic environment. Sci Total Environ, 225**,** 109-118.

ICPR 2015. Internationally Coordinated Management Plan 2015 for the International River Basin District of the Rhine. Koblenz, Germany: (ICPR), I. C. f. t. P. o. t. R.

IT.NRW. 19.12.2018 2019. RE: Agricultural census 2016 about livestock and livestock densities in North-Rhine-Westphalia (Agrarstrukturerhebung 2016 zu Viehhaltungen und Viehbeständen in Nordrhein-Westfalen). Place: Central statistical and IT services provider of North Rhine-Westphalia. Type to Niebaum, G.

Jelić, A., Gros, M., Petrović, M., et al. 2012. Occurrence and Elimination of Pharmaceuticals During Conventional Wastewater Treatment. In: Guasch, H., Ginebreda, A. & Geiszinger, A. (eds.) Emerging and Priority Pollutants in Rivers: Bringing Science into River Management Plans. Berlin, Heidelberg: Springer.

Karfusehr, C., Kayser, A., te Gempt, R., et al. 2018. Concentrations and sources of antibiotics in regions with intensive livestock production in Lower-Saxony. Grundwasser, 24**,** 3-11.

Kay, P., Blackwell, P. A. & Boxall, A. B. 2005. A lysimeter experiment to investigate the leaching of veterinary antibiotics through a clay soil and comparison with field data. Environ Pollut, 134**,** 333-41.

Kim, K.-R., Owens, G., Kwon, S.-I., et al. 2010a. Occurrence and Environmental Fate of Veterinary Antibiotics in the Terrestrial Environment. Water, Air, & Soil Pollution, 214**,** 163-174.

Kim, S. C., Davis, J. G., Truman, C. C., et al. 2010b. Simulated rainfall study for transport of veterinary antibiotics--mass balance analysis. J Hazard Mater, 175**,** 836-43.

Kim, Y., Lim, S., Han, M., et al. 2012. Sorption characteristics of oxytetracycline, amoxicillin, and sulfathiazole in two different soil types. Geoderma, 185-186**,** 97-101.

Kivits, T., Broers, H. P., Beeltje, H., et al. 2018. Presence and fate of veterinary antibiotics in age-dated groundwater in areas with intensive livestock farming. Environmental Pollution, 241**,** 988-998.

Kümmerer, K., Schuster, A., Längin, A., et al. 2011. Identification and und assessment of selected pharmaceuticals and their metabolites (degradation and transformation products) in the water cycle (Identifizierung und Bewertung ausgewählter Arzneimittel und ihrer Metaboliten (Ab- und Umbauprodukte) im Wasserkreislauf). Dessau-Roßlau, Germany: German Environment Agency (UBA).

Lahr, J., Bondt, N., Koeijer, d. T., et al. 2017. A step towards the environmental prioritisation of veterinary medicines from animal manure. Water Matters: Knowledge Journal for Water Professionals.

Leenstra, F., Vellinga, T., Neijenhuis, F., et al. 2014. Manure: A valuable resource. Wageningen, the Netherlands: Research, W. U. L. & commissioned by the Dutch Ministries of Economic Affairs and Infrastructure and the Environment.

Lienert, J., Güdel, K. & Escher, B. I. 2007. Screening Method for Ecotoxicological Hazard Assessment of 42 Pharmaceuticals Considering Human Metabolism and Excretory Routes. Environmental Science Technology, 41**,** 4471-4478.

Lindberg, R. H., Wennberg, P., Johansson, M. I., et al. 2005. Screening of human antibiotic substances and determination of weekly mass flows in five sewage treatment plants in Sweden. Environ Sci Technol, 39**,** 3421-9.

LSN 2019. LNS online regional database (LSN Online Regionaldatenbank). Hannover, Germany: Statistical Office Lower Saxony.

Mackay, N., Mason, P. & Di Guardo, A. 2005. VetCalc Exposure Modelling tool for Veterinary Medicines. UK: Cambridge Environmental Assessments, Veterinary Medicines Directorate.

Mauer, C. 2011. Technical and economical aspects on seperat treatment of hospital wastewaters (Technische und ökonomische Aspekte der separaten Erfassung und Behandlung von Krankenhausabwasser). PhD Thesis, Technische Hochschule Aachen, Aachen, Germany.

Maurer, M., Escher, B. I., Richle, P., et al. 2007. Elimination of beta-blockers in sewage treatment plants. Water Res, 41**,** 1614-22.

Moffat, A. C., Osselton, M. D. & Widdop, B. 2011. Part 2: Monographs. In: Moffat, A. C., Osselton, M. D. & Widdop, B. (eds.) Clarke's Analysis of Drugs and Poisons: Fourth edition. London: Pharmaceutical Press.

Montforts, M. 1999. Environmental risk assessment for veterinary medicinal products: Part 1. Other than GMO-containing and immunological products. First update. Bilthoven, the Netherlands: National Institute of Public Health and the Environment.

OECD 2015. Environment at a glance: OECD indicators. Paris, France: OECD Publishing.

Oosterhuis, M., Sacher, F. & Ter Laak, T. L. 2013. Prediction of concentration levels of metformin and other high consumption pharmaceuticals in wastewater and regional surface water based on sales data. Sci Total Environ, 442**,** 380-8.

Orias, F. & Perrodin, Y. 2013. Characterisation of the ecotoxicity of hospital effluents: a review. Sci Total Environ, 454-455**,** 250-76.

Ostermann, A., Siemens, J., Welp, G., et al. 2013. Leaching of veterinary antibiotics in calcareous Chinese croplands. Chemosphere, 91**,** 928-34.

Pan, M. & Chu, L. M. 2016. Adsorption and degradation of five selected antibiotics in agricultural soil. Sci Total Environ, 545-546**,** 48-56.

Pan, M. & Chu, L. M. 2017. Leaching behavior of veterinary antibiotics in animal manure-applied soils. Sci Total Environ, 579**,** 466-473.

Paut Kusturica, M., Tomas, A. & Sabo, A. 2017. Disposal of Unused Drugs: Knowledge and Behavior Among People Around the World. In: de Voogt, P. (ed.) Reviews of Environmental Contamination and Toxicology. Switzerland: Springer.

Persson, M., Sabelstrom, E. & Gunnarsson, B. 2009. Handling of unused prescription drugs--knowledge, behaviour and attitude among Swedish people. Environ Int, 35**,** 771-4.

Radjenovic, J., Petrovic, M. & Barcelo, D. 2009. Fate and distribution of pharmaceuticals in wastewater and sewage sludge of the conventional activated sludge (CAS) and advanced membrane bioreactor (MBR) treatment. Water Res, 43**,** 831-41.

Ray, P., Chen, C., Knowlton, K., et al. 2017. Fate and effect of antibiotics in beef and dairy manure during static and turned composting. J Environ Qual, 46**,** 45-54.

Rosal, R., Rodriguez, A., Perdigon-Melon, J. A., et al. 2010. Occurrence of emerging pollutants in urban wastewater and their removal through biological treatment followed by ozonation. Water Res, 44**,** 578-88.

Sacher, F. & Thoma, A. 2014. Inventory of micro pollutants in surface waters in Baden Wuerttemberg (Spurenstoffinventar der Fließgewässer in Baden-Württemberg). Stuttgart, Germany: Ministry of the Environment, Climate Protection and the Energy Sector Baden-Württemberg.

Santonja, G. G., Georgitzkis, K., Scalet, B. M., et al. 2017. Best Available Techniques (BAT) Reference Document for the Intensive Rearing of Poultry or Pigs: Industrial Emissions Directive 2010/75/EU (Integrated Pollution Prevention and Control). European Commission.

Song, W. & Guo, M. 2014. Residual Veterinary Pharmaceuticals in Animal Manures and Their Environmental Behaviors in Soils. In: He, Z. & Zhang, H. (eds.) Applied Manure and Nutrient Chemistry for Sustainable Agriculture and Environment. Springer Science and Business.

Spielmeyer, A., Ahlborn, J. & Hamscher, G. 2014. Simultaneous determination of 14 sulfonamides and tetracyclines in biogas plants by liquid-liquid-extraction and liquid chromatography tandem mass spectrometry. Anal Bioanal Chem, 406**,** 2513-24.

Ternes, T. A., Bonerz, M., Herrmann, N., et al. 2007. Irrigation of treated wastewater in Braunschweig, Germany: an option to remove pharmaceuticals and musk fragrances. Chemosphere, 66**,** 894-904.

UBA. 2018. Treatment of animal manure (Behandlung von Wirtschaftsdüngern) [Online]. Dessau-Roßlau, Germany: German Environment Agency (UBA). Available: https://www.umweltbundesamt.de/behandlung-von-wirtschaftsduengern#Landwirtschaft [Accessed 16.06.2019 2019].

Van Geijlswijk, I. M., Heederik, D. J. J., Mouton, J. W., et al. 2018. Usage of Antibiotics in Agricultural Livestock in the Netherlands in 2017: Trends and benchmarking of livestock farms and veterinarians. Utrecht, the Netherlands: Netherlands Veterinary Medicines Institute (SDa).

Veldman, K. T., Mevius, D. J., Wit, B., et al. 2018. Maran 2018: Monitoring of Antimicrobial Resistance and Antibiotic Usage in Animals in the Netherlands in 2017. Lelystad, the Netherlands: Wageningen Bioveterinary Research Food and Consumer Product Safety Authority; National Institute for Public Health and the Environment; Netherlands Veterinary Medicines Institute.

Vieno, N., Tuhkanen, T. & Kronberg, L. 2007. Elimination of pharmaceuticals in sewage treatment plants in Finland. Water Research, 41**,** 1001-1012.

Wallmann, J. 2017. RE: Veterinary sales data for post codes 48 and 49. Place: Federal Office of Consumer Protection and Food Safety (BVL),. Type to Wöhler, L.

Wallmann, J., Bode, C., Bender, A., et al. 2018. Antibiotic sales in Germany 2017 (Abgabemengenerfassung von Antibiotika in Deutschland 2017). Deutsches Tierärzteblatt, 66.

Wang, Q. & Yates, S. R. 2008. Laboratory Study of Oxytetracycline Degradation Kinetics in Animal Manure and Soil. Journal of Agricultural and Food Chemistry, 56**,** 1683-1688.

Wang, Q. Q., Bradford, S. A., Zheng, W., et al. 2006. Sulfadimethoxine degradation kinetics in manure as affected by initial concentration, moisture, and temperature. J Environ Qual, 35**,** 2162-9.

Weinfurtner, K. 2011. Matrix parameters and storage conditions of manure Dessau-Roßlau, Germany: German Environment Agency (UBA).

West, L. M., Diack, L., Cordina, M., et al. 2014. A systematic review of the literature on 'medication wastage': an exploration of causative factors and effect of interventions. Int J Clin Pharm, 36**,** 873-81.

Yang, X., Flowers, R. C., Weinberg, H. S., et al. 2011. Occurrence and removal of pharmaceuticals and personal care products (PPCPs) in an advanced wastewater reclamation plant. Water Res, 45**,** 5218-28.

Zorita, S., Martensson, L. & Mathiasson, L. 2009. Occurrence and removal of pharmaceuticals in a municipal sewage treatment system in the south of Sweden. Sci Total Environ, 407**,** 2760-70.

Zuehlke, S., Duennbier, U., Lesjean, B., et al. 2006. Long-Term Comparison of Trace Organics Removal Performances Between Conventional and Membrane Activated Sludge Processes. Water Environment Research, 78**,** 2480-2486.

1. IQVIA is an Institute for Human Data Science. Data retrieved from IQVIA is based on drug prescriptions that are retailed by pharmacies. Sales data for OTC-compounds are included by extrapolating data from pharmacy questionnaires. [↑](#footnote-ref-1)
2. SFK is the Dutch Foundation of Pharmaceutical Statistics and collects pharmaceutical sales data for 95% of the Dutch community pharmacies. The data retrieved from SFK include an extrapolation to all pharmacy pharmaceutical sales. According to SFK, an additional 7% of pharmaceutical sales are not included in the data by SFK because these drugs are dispersed by private practitioners. We accounted for this by an increase of pharmacy sales by 7%. [↑](#footnote-ref-2)
3. Assuming that the category “other poultry farming sectors” exclusively includes the chicken farming sector. [↑](#footnote-ref-3)
4. All veal were counted as beef cattle. [↑](#footnote-ref-4)
